# Progress in Diamanes and Diamanoids Nanosystems for Emerging Technologies

**DOI:** 10.1002/advs.202105770

**Published:** 2022-02-17

**Authors:** Santosh K. Tiwari, Raunak Pandey, Nannan Wang, Vijay Kumar, Olusegun J. Sunday, Michał Bystrzejewski, Yanqiu Zhu, Yogendra Kumar Mishra

**Affiliations:** ^1^ Faculty of Chemistry University of Warsaw 1 Pasteur Str. Warsaw 02‐093 Poland; ^2^ Key Laboratory of New Processing Technology for Nonferrous Metals and Materials Ministry of Education School of Resources Environment and Materials Guangxi University Nanning 530600 China; ^3^ Department of Chemical Science and Engineering Kathmandu University Dhulikhel 44600 Nepal; ^4^ Department of Physics National Institute of Technology Srinagar Hazratbal Jammu and Kashmir 19006 India; ^5^ Department of Physics University of the Free State P.O. Box 339 Bloemfontein ZA9300 South Africa; ^6^ College of Engineering Mathematics and Physical Sciences University of Exeter Exeter EX4 4QF UK; ^7^ Smart Materials NanoSYD Mads Clausen Institute University of Southern Denmark Alsion 2 Sønderborg 6400 Denmark

**Keywords:** diamane, diamanoids, quantum electronics application and new devices, structural properties, synthesis

## Abstract

New materials are the backbone of their technology‐driven modern civilization and at present carbon nanostructures are the leading candidates that have attracted huge research activities. Diamanes and diamanoids are the new nanoallotropes of sp^3^ hybridized carbon which can be fabricated by proper functionalization, substitution, and via Birch reduction under controlled pressure using graphitic system as a precursor. These nanoallotropes exhibit outstanding electrical, thermal, optical, vibrational, and mechanical properties, which can be an asset for new technologies, especially for quantum devices, photonics, and space technologies. Moreover, the features like wide bandgap, tunable thermal conductivity, excellent thermal insulation, etc. make diamanes and diamanoids ideal candidates for nano‐electrical devices, nano‐resonators, optical waveguides, and the next generation thermal management systems. In this review, diamanes and diamanoids are discussed in detail in terms of its historical prospect, method of synthesis, structural features, broad properties, and cutting‐edge applications. Additionally, the prospects of diamanes and diamanoids for new applications are carefully discussed. This review aims to provide a critical update with important ideas for a new generation of quantum devices based on diamanes and diamanoids which are going to be an important topic in the future of carbon nanotechnology.

## Graphical abstract

Diamanes and diamanoids are newly discovered graphitic carbon nanostructures. These nanosystems are sp^3^ carbon networks along with few sp^2^ hybridized carbon atoms which can be fabricated under controlled temperature & pressure using graphitic system as a precursor. These materials exhibit extraordinary properties, which could be an asset for new research in fundamental physics, quantum devices, photonics, and space research.

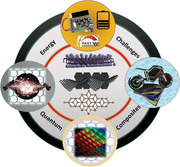

## 1 Introduction

**Figure 1:**
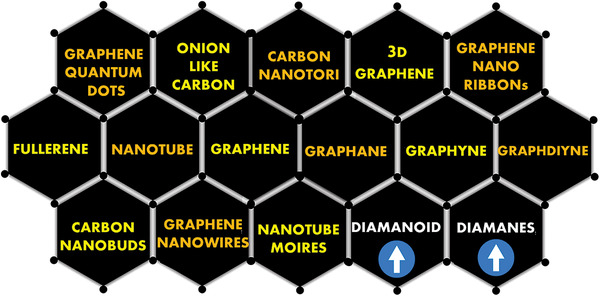
Different forms of carbon nanoallotropes, including diamane and diamanoids.

Diamane (sp^3^ bonded two crystalline carbon layers) and diamanoids (composite of diamane and graphene), the two new nanomaterials which have been incorporated into the carbon family, have caught the attention of the scientific community. The stability and existence of these materials were first predicted by Chernozatonskii et al. in 2009,^[^ ^1^ ^]^ but during the last 5 years, immense developments of these materials have been witnessed. Several experimental routes have been discovered for the synthesis of diamane and diamanoids which are based on the results of computational and theoretical studies.^[^ ^1^ ^]^ Thus, diamane and diamanoids are those fascinating nanocarbons which could be a new class of sensational materials similar to those such as MAX phases, single‐layer hexagonal boron nitride (hBN), metal dichalcogenides, and graphene. Looking back, as the precursor of diamane and diamanaoids, carbon nanomaterials are playing a crucial role in our modern civilization and it is hard to imagine the current commercial aspects of the internet,^[^ ^2^ ^]^ mobile phone,^[^ ^3^ ^]^ optical fiber,^[^ ^4^ ^]^ pharma‐biomedical,^[^ ^5^ ^]^ space,^[^ ^6^ ^]^ defense,^[^ ^6^ ^]^ automobile,^[^ ^7^ ^]^ and textile industries^[^ ^8^ ^]^ without carbon materials. According to Buckley et al.,^[^ ^9^ ^]^ carbon‐based materials will replace majority of non‐carbonaceous semiconductor materials used in various high technologies, including supercomputers and the next generation communication devices by 2040. Such tremendous growth and demanding applications of carbon materials are owing to their tunable physical properties and exceptional bonding capabilities.^[^ ^10^ ^]^ That is why, as an element, carbon exhibit leading functionalities over other elements in the periodic table, being the most prominent candidate for nanoscience and advanced technologies.^[^ ^11^ ^]^ According to the Scopus database, almost (30–35)% of the total scientific publications are directly or indirectly associated with carbon‐based materials.^[^ ^12^ ^]^ Thus, carbon materials research is dominating the entire scientific research activities for various industrial applications. Referring to the early exploration of carbon nanomaterials, 1985 and onwards were the years that ignited the experimental nanotechnology and carbon materials engineering. In this regard, in 1985, fullerene (OD, **Figure** **1**) was discovered by Kroto et al.^[^ ^12^, ^13^ ^]^ which opened the excitement and probabilities of nano‐allotropes of carbon.^[^ ^13^ ^]^ The discovery of fullerene was a sensation, and within 10 years of its discovery, reports on applications of fullerene especially for biomedical and bioimaging,^[^ ^14^ ^]^ drug delivery,^[^ ^15^ ^]^ HIV treatment,^[^ ^16^ ^]^ and enhancement of thermo‐mechanical properties of polymers,^[^ ^17^ ^]^ were developed and accordingly utilized. The extensive research around the globe on fullerene and fullerene‐like (carbon onion and higher fullerenes) nanomaterials led to the discovery of carbon nanotubes (CNTs, 1D, Figure 1) which was first reported by Ijima et al. in 1992.^[^ ^18^ ^]^ This was another unique carbon atom‐based nanomaterials with outstanding thermo‐mechanical strength, very high chemical inertness, great flexibility, lightweight, large surface area, and excellent electronic conductivity.^[^ ^19^, ^20^, ^21^ ^]^ Consequently, different types of nanotubes have been fabricated, including several forms of inorganic nanotubes and many of which are part of modern technologies of the 21st century.^[^ ^22^ ^]^ Till date, various routes (arc‐discharge, chemical vapor deposition, hydrothermal, chemical exfoliation, electrochemical expansion, etc.) have been developed for the synthesis of desired fullerene and CNTs. Further extension of the CNT arena has been documented as several new forms, such as nano buds, nano horns, carbon dots, etc. as shown in Figure 1 along with other carbon nano allotropes.^[^ ^23^, ^24^ ^]^ It is noted that surface modification and functionalization of fullerene, CNTs, and their derivatives are very difficult, which limits their full potentials toward real applications. Therefore, some of these materials are under‐explored.^[^ ^25^ ^]^ Transitioning from CNTs to graphene, in 2004, Andre Geim et al. successfully achieved a single layer of carbon atoms arranged in a 2D honeycomb lattice (Figure 1) and named it graphene.^[^ ^12^, ^26^ ^]^ It is one of the most studied nanomaterials in the current scenario owing to its great prospects. Graphene is often called "a magical material" due to its broad potentials for applications in almost every field of science and technology. Physical properties of the graphene‐like structures including ultra‐high surface area, exceptional mechanical strength, great chemical inertness, unique quantum hall effect, and unusual band structure make them an ideal candidate for the next generation applications from computing to space research programs.^[^ ^26^, ^27^, ^28^, ^29^ ^]^ Such structures exist in several forms (Figure 1), such as nanoribbons, graphene quantum do ts, etc., with revised properties that can be tailored for emerging applications. The details of graphene and its derivatives have been widely discussed.^[^ ^26^, ^27^, ^28^, ^29^ ^]^ Currently, thousands of potential applications of the aforementioned nanocarbons have been proposed and more than 1000 review articles have been documented in this special research domain. However, till date, only a few research articles and scientific commentary are associated with diamane and diamanoids. Therefore, a comprehensive review on this specific subject is needed, to highlight their huge potentials and future directions. Based on the state‐of‐the‐art reports, this review will provide an in‐depth coverage of the properties, synthesis, and application of diamane and diamanoids, with comprehensive references for further reading. Herein, we have particularly focused on technological applications and related devices for which diamane and diamanoids could be utilized as the best alternative materials. The authors believe that the vast amount of scientific data, new ideas with simple explanations, several schematic diagrams, and key future challenges will provide a solid platform for readers to get a firm grip on the attractive wonders of diamane and diamanoids.

## 2 Early Works on Diamane and Diamanoids

In 1994, the International Union of Pure and Applied Chemistry (IUPAC) introduced the term “graphene” which provided the nomenclature, reactions, and terminology of graphene and related nanosystems.^[^ ^30^ ^]^ The name was just an introduction at that time when no proper synthesis was attained, although initial work on graphene was carried out in as early as 1853.^[^ ^12^, ^31^ ^]^ The nomenclature provided by IUPAC proliferated the interest of researchers and scientists to develop 2D structures. Although graphene for the first time was theoretically discovered in 1947 by P. R. Wallace, the identification and isolation through experiment was done several decades later. In 2004, for the first time, graphene was synthesized/isolated experimentally by the research group of A. Geim and K. Novoselov.^[^ ^26^, ^27^ ^]^ In 2007, Sofo et al. isolated the single layer graphene through several studies, followed by hydrogenation to the single‐layered structure, and evaluated the properties of these new structures.^[^ ^32^ ^]^ These early works a platform for obtaining 2D structures with good stability in hydrogenated form. The race of finding new 2D structures on top of graphene, thus, began. In 2009, Elias et al.^[^ ^33^ ^]^ partially hydrogenated the single‐layer graphene to form a reversible structure which exhibited enhanced electronic and transport properties. In the same year, Chernozatonskii et al. theoretically studied the behavior of bilayer graphene.^[^ ^1^ ^]^ In this study, bilayer graphene was hydrogenated to synthesize hydrogenated bilayer graphene named “diamane.” Stacking sequences of bilayer graphene in AB and AA sequence were utilized to be hydrogenated to create “diamane” and its isomer “diamane II,” respectively.^[^ ^1^ ^]^ These early developments initiated the research on diamanes, including exploration of their various physical, chemical, mechanical, and thermal properties. In 2012, Jones et al. hydrogenated the trilayer graphene to form a new structure called triamane.^[^ ^34^ ^]^ The hydrogenation of layered graphene provided various properties by the use of other functional groups. In this line, Bakhrav et al. studied (2020) the possibility of fluorinated diamond (F‐diamane), and eventually obtained a structure with carbon‐carbon interlayer bonding by fluorination of bilayer graphene for the first time.^[^ ^35^ ^]^ However, the experimental formation of diamane was conducted not before 2018.^[^ ^36^ ^]^ Other functional groups of chlorine and hydroxyl were also added by other scientists to tune the properties of diamane. Interestingly, during the last (5–6) years, bilayer graphene was substituted by atoms of nitrogen, boron, lithium, etc., to tune the properties for wide range applications. In addition, twisted bilayer graphene was also hydrogenated to obtain Moire's pattern on diamane with unique properties.

A hypothetical assumption was made for the formation of diamanoids from non‐supported few‐layer graphene in hydrogen cold plasma until 2019. In the same year, Piazza et al. synthesized the hydrogenated few‐layer graphene, called “diamanoids,” inside the hot filament reactor to form a stable sp^3^ structure.^[^ ^37^, ^38^ ^]^ In summary, we can say that intensive experimental and theoretical work on graphene and its derivative between 2000 to 2010 were mainly focused on the evaluation of diamane and diamanoids.

## 3 Diamane and Diamanoids

**Figure 2:**
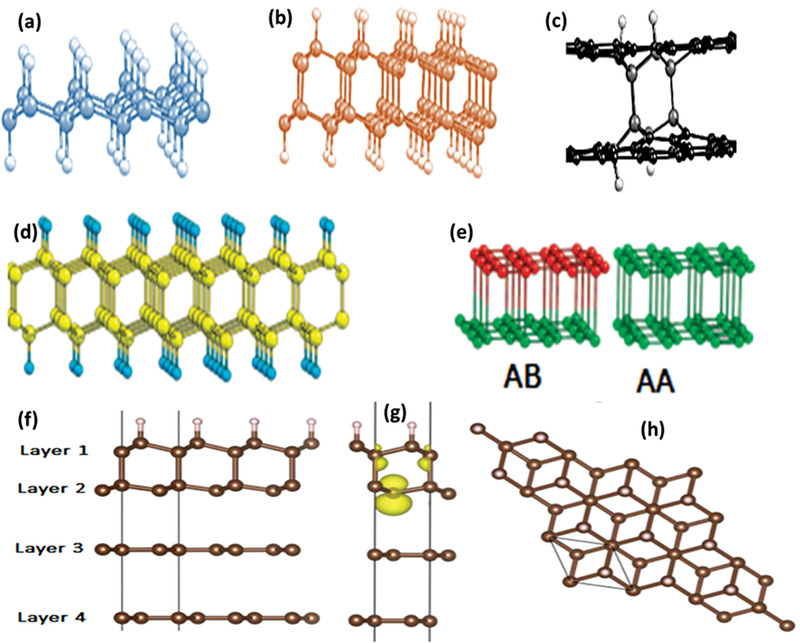
a,b) Structural units of diamane in different configurations. c) Schematic presentation of the diamane nucleus formation owing to the hydrogenation of bilayer graphene (hydrogen atoms that are attached on the two sides initiated the stacking of the carbon atoms over each other). The carbon and hydrogen atoms are shown in dark and bright gray, respectively, in (a), while black circles in (c) correspond to the sp^2^ carbon atoms. Reproduced under the terms of the Creative Commons CC‐BY license.^[^ ^50^ ^]^ Copyright 2021, TheAuthors. Published by MDPI. d,e) Molecular geometry of quasi 2D carbon nanofilms of diamane (AB) with AB stacking of two layers. Carbon atoms are marked by yellow (light gray); hydrogen is marked by blue (gray). Reproduced with permission.^[^ ^51^ ^]^ Copyright 2010, American Chemical Society. f,g) The two side views, and h) top view of the partially hydrogenated few‐layer graphene used in DFT calculations with an ABBA stacking. Reproduced with permission.^[^ ^38^ ^]^ Copyright 2020, Elsevier.

In the course of exploring new 2D structures, scientists came across 2D graphene‐based materials termed as “diamane” and “diamanoid” which exhibit superb potentials for applications in biomedical, sensory, electrical, nano‐electronics, and other most important sectors of quantum computing technology. Diamane is termed as a double‐layered diamond or graphene bearing the characteristics of the thinnest hard material. Bilayer graphene of sp^2^ hybridization is converted to sp^3^ hybridized structure through covalent bonding strategies. In diamane, two carbon atoms of two subatomic lattices with hydrogen atoms form the C_2_H layered unit, while the other non‐attached carbon layer forms a covalent bond to the sub‐lattices of other carbon atoms of neighboring graphene, thereby leading to the formation of a new carbon system, called diamane with a structure shown in **Figure** **2**a–c.^[^ ^1^ ^]^ The two lone pairs of electron on the surface of the carbon layer, however, remain undisturbed. Doing so, CCCC stacking in the vertical direction (in the form of 111 stacking) is prevalent until four carbon atoms are placed in a unit cell, leading to the formation of diamane.^[^ ^39^, ^40^ ^]^ The hybridized layer of diamond or lonsdaleite exhibits hexagonal or cubic lattice (Figure 2b), provided that half of the carbon is hydrogenated, and the other half is bonded to the neighboring carbon layers (Figure 2b). Even though all these processes occur just at a very small nanometer scale, the final product is important on a much greater scale.^[^ ^1^, ^36^, ^37^, ^41^ ^]^ The name of “diamane” was coined in analogy to the “graphene–graphane,” as it is virtually similar to a diamond‐like structure renovated from bilayer graphene and hydrogen, as discussed above.^[^ ^42^, ^43^ ^]^ Other names for diamane such as interlayer‐bonded bilayer graphene, hydrogenated few‐layer graphene, diamandol, diamondene, hydrogenated diamond, and hydrogenated bilayer graphene have also been used by different research groups.^[^ ^44^, ^45^, ^46^ ^]^ The formation of diamane requires stacking of bilayer graphene in two of the following sequences: AB (Bernal) stacking and AA (Lonsdaleite) stacking (Figure 2d,e). The C_2_H unit of diamane has an AB stacking sequence and the isomer of diamane (diamane‐II) has an AA stacking sequence, which strongly affects its physical and chemical properties.^[^ ^1^, ^45^, ^47^, ^48^ ^]^ The AA stacked chain isomers of graphene are the most stable structure, and under pressure it lays the foundation of 3D graphene crystals in hydrogenated form as well.^[^ ^49^ ^]^ With this configuration, genuine diamane is formed, but if the bilayer graphene is twisted to a certain angle normally up to 30˚, Moire's pattern is formed on the graphene layers.^[^ ^47^, ^50^ ^]^ These twisted layers again could form new structures upon further functionalizations.

In a pristine form and a fully free state, atomically thin diamond film is unstable, due to the lack of thermally stable structures in this 2D system, as per the laws of physics. Thus, various hydrocarbon, hybridizations, and functional moieties are used to fabricate the stable diamane structure and to attain its thermodynamic stability. That is why in recent years, hydroxyl, fluorine, oxygen, nitrogen, and other functional groups are attached to achieve different hybrid structures of diamanes.^[^ ^35^, ^52^, ^53^ ^]^ In addition to the functional groups, pressure can also be applied as an alternative for the formation of diamanes, but maintaining stability at low pressure is challenging. Pressure application for the formation of diamane is normally discouraged due to the complex method of synthesis and the need for pressure.^[^ ^35^, ^52^, ^53^ ^]^ The other way to achieve various kinds of diamane is the substitution of atoms as dopants in graphene, and stabilizing the hybridization to form modified diamanes. During the last 5 years, nitrogen, boron, sulfur, and lithium atoms have already been used as dopants, but explorations of other dopants are also developing rapidly.^[^ ^44^, ^54^, ^55^ ^]^ These methods bring in new opportunities for discovering exciting properties of diamanes, which are very important for quantum electronics and the next generation energy storage devices.

When the number of hydrogenated graphene layers is increased from two to a few layers (3–5), a new 2D structure of carbon with entirely different properties is formed and commonly called diamanoid (partially hydrogenated few layers graphene. Figure 2f,g). The DFT calculated geometry of diamanoid with an ABBA stacking is presented in Figure 2h for a better understanding of the bonding patterns. Initially, the hydrogen radicals produced by the low temperature and pressure in a hot filament process were used for the synthesis of diamanoid, but new fabrication techniques have recently been developed that were capable of producing diamanoids having different layers.^[^ ^36^, ^38^, ^55^ ^]^ In the hot filament process, diamanoid was fabricated by hydrogenating the top carbon layer, whilst the bottom layer is covalently bonded with the neighboring graphene layer, as depicted in Figure 2f,g.

The Bernal stacking is frequently observed in diamane, but not in diamanoid due to its structural instability. But other different types of stacking sequences such as (ABB) diamanoids, (ABBA) diamanoids, and (AABBCC) diamanoids, will all favor the formation of diamanoids. However, the nomenclature of few‐layered (3–5) graphene does not specify up to how many layers should it be considered as ‘a few’, hence, the maximum allowed number of layers used in diamanoid synthesis has yet to be finalized along with more intensive research.^[^ ^37^ ^]^ Lately, some diamanoids with fluorine and hydrogen have been reported, however these reports did not attract sufficient attention from the scientific community, owing to thermodynamic reasons.^[38]^ In addition, most of the studies carried out on diamane, diamanoids, and carbon‐like thin films are theoretical and simulation‐based computational methods at present. Therefore, there is a great room for experimentations. Significant experimental works (e. g. synthesis, characterization, and applications) on diamane and diamanoids will be discussed in the next sections.

### 3.1 Properties of Diamane

#### 3.1.1 Structure

**Figure 3:**
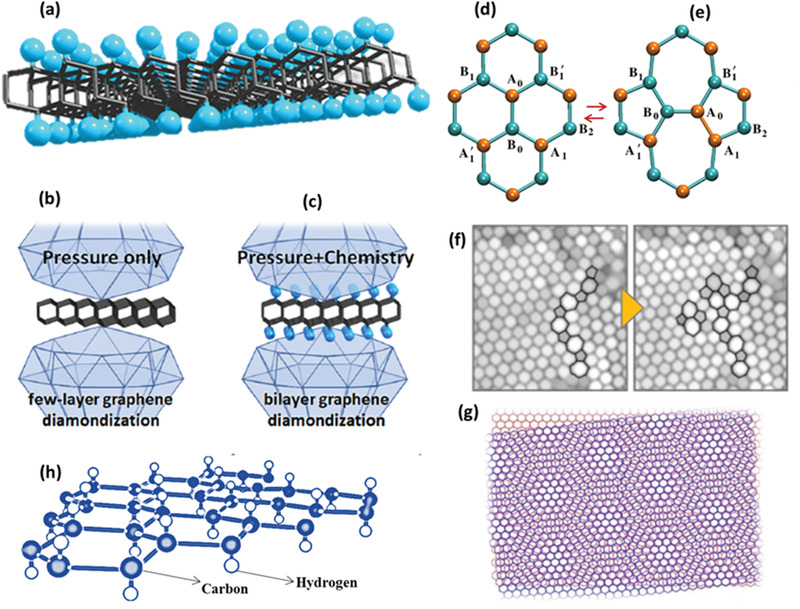
a) Arrangement of carbon atoms and layers in diamanes. b) Diamondization phenomenon in the case of 3‐, 4‐, 6‐,12‐, and 7‐ layered graphene under applied pressure alone, but it is not possible in the case of bilayered graphene. c) Use of pressure along with surface manipulation (chemistry) can cause bilayered graphene diamondization by water as a pressure transition medium. a‐c) Reproduced with permission.^[^ ^61^ ^]^ Copyright 2021, American Chemical Society. d,e) The equilibrium structures of Stone–Wales defect in the case of pristine graphane which also occurs in diamanes and diamanoids, as a characteristics. Reproduced with permission.^[^ ^34^ ^]^ Copyright 2013, IOP Publishing, Ltd. f) AC‐HRTEM images showing Stone–Wales transformations in graphene and 2D‐related systems. Reproduced with permission.^[^ ^28^ ^]^ Copyright 2013, Macmillan Publishers Limited. g) The Moire's pattern is a characteristics of twisted bilayered graphene, which also exists in diamanes and diamanoids (image credit: Andrei/Rutgers University‐New Brunswick). h) Molecular structure of graphane, a common component for diamanes and diamanoids.

Diamane synthesized from bilayer graphene has offered the opportunities to prove its potentials in nanotechnology and the next generation of nanodevices. Because of the structural change in graphitic carbon skeleton, it is prone to alter the physical and chemical properties, especially electronic properties. The changes in structure resulting in changes in electrical, optical, mechanical, and thermal properties will be discussed in this section. The uniqueness of these structures largely depends on the type of structural deformation, the amount of sp^3^ carbon, the nature of hetero atoms, and the area of hydrogenated graphene.^[^ ^56^, ^57^, ^58^, ^59^, ^60^, ^61^ ^]^ Thus, the primary factor is to select proper functional groups in terms of their molecular or atomic states to form the desired structures in diamane. The selection of the fittest form of hydrogen in the crystal lattice of diamane is illustrated in **Figure** **3**a.^[^ ^61^, ^62^, ^63^ ^]^ In this regard, it is observed that uniform adsorption of atomic hydrogen to bilayer graphene leads to a strong chemical bonding between the layers of graphene, which, in turn, greatly stabilizes the structure of diamane, owing to the removal of Van der Waal forces.^[^ ^64^ ^]^ Molecular hydrogen could only be used to perform physisorption on the surface of graphene rather than forming conventional bonds as discussed previously.^[^ ^64^ ^]^ Furthermore, the proper selection of atoms or molecules can change the configuration for graphene layers, and consequently diamane with different peculiarities can be generated. Based on the content of hydrogen, stacking pattern, and configuration, the structure of diamane can be classified as Type‐I, fully interlayer bonded configuration from AB or AA (Figure 2e) stacked bilayers with half hydrogen coverage, and the non‐functionalized carbon atom bonded to a neighboring layer; Type‐II, hybrid of the sp^2^–sp^3^ system formed from AB stacked bilayer, seen as continuous “stripes” of pristine bilayered graphene with stripes of fully interlayer bonded Type I‐like domain; and Type III exhibiting super‐lattices of finite diamond‐like interlayer bonded domains embedded within the graphene planes and mainly consisting of three twist angles (6˚, 9.43˚, and 13.17˚), which is also called as twisted bilayer graphene. The grouping of diamane has made it easy for understanding their stereo‐chemical properties.^[^ ^65^ ^]^ Viewing the morphology of this 2D nanosystem, an octahedral morphology is revealed (Figure 3a) where two outermost layers were delaminated and partially graphitized, resulting in a non‐symmetrical reconfiguration, while the central layers were kept in the original diamond configurations. This structure is very close to the symmetry and morphology of those reported recently by Li et al. for the diamondization of a few layered and bi‐layered graphene (Figure 3b,c).^[^ ^56^, ^61^ ^]^ Experimentally, it is proved that hexagonal and rhombohedral configurations are the most stable forms (thermodynamic stable) of diamane, owing to the hexagonal Bernal stacking and lower energy than other stacking patterns. These two configurations are very similar to those cases of Stone–Wales defect in graphene (Figure 3d,e), but not identical to other 2D systems. The Stone–Wales defect in graphene and related systems are shown in the HRTEM image (Figure 3f), as described elsewhere.^[^ ^40^, ^56^, ^61^ ^]^ The schematic structure of graphene, a key component of diamanes and diamanoids, is shown in Figure 3h, for comparison, even though all the three are entirely dissimilar in nature.^[^ ^56^, ^57^, ^58^, ^59^, ^60^, ^61^, ^63^ ^]^ The rhombohedral sequential layer has also been reported in the case of conversion of multilayered graphene to ultra‐thin diamond films via chemical functionalization.^[^ ^57^, ^58^, ^59^, ^60^, ^61^ ^]^ Similarly, cubic and hexagonal structures can be achieved when AB stacked and AA stacked diamanes are synthesized; whilst diamane with Moire's pattern (a set of straight or curved lines being superposed onto another set), that is similar to graphene (but not identical) (Figure 3g) from rotational stacking, can be achieved using pristine graphene with a smaller twist angle between layers.^[^ ^55^, ^56^, ^61^, ^64^, ^66^ ^]^

The relative arrangement of the carbon layer is more important than the thickness parameter in the case of diamane, along with AB stacking being more significant than the AA stacking.^[^ ^58^ ^]^ This explains why the strength and stability of diamane is more than that of graphane and graphene which are further determined by increased stiffness coefficient. Hence, the fabrication of diamane from more than two graphene layers is possible, however it requires harsh pressure and temperature conditions.^[^ ^1^ ^]^ The lowest energy per atom is exhibited by AB and ABC stacked diamanes, which accounts for the different structures of diamanes.^[^ ^31^, ^48^, ^59^ ^]^

To avoid misperception, it is necessary to point out that diamond nanocrystals are different from diamanes, because of the hybridization of carbon atoms and interlayer CAC stacking pattern within the structural domains.^[^ ^65^ ^]^ Correspondingly, three alternating graphene layers in ABC stacking form cubic diamond, and on the other hand, two alternating graphene layers in AA stacking produces hexagonal diamond.^[^ ^60^ ^]^ These patterns could play a crucial role in distinguishing the different structures of diamanes after the removal of functional groups in functionalized diamanes, and in diamanes synthesized using pressure. Thus, orbital mixing is the key driving force for the transformation of bilayered graphene into diamane, and functionalization greatly increases the orbital mixing owing to the uniform electron cloud distribution. This is why the removal of functional moieties from the surface of diamane badly destabilizes its structures.^[^ ^31^, ^59^ ^]^

External factors, including temperature, pressure, irradiation, and surface modification via covalent interactions, can be used to create imperfection in the structure of diamane by the graphitization process. These imperfections are originated from the energy preference of graphite over diamond, leading to changes in physical characteristics and thermodynamic stability. Bulk diamonds are metastable due to the action of the transformation barrier. An increase in phase transition pressure for graphene–diamane conversion and decrease or disappearance of stability of thinnest films could be due to the shift in phase equilibrium if the passivation of hydrogen or fluorine was not carried out appropriately. In this way, the passivation of diamond structure for stability is mandatory prior to converting the pristine surfaced diamane films.^[^ ^61^ ^]^ Stress and strain are also expected once layers are hydrogenated along with grain boundaries between diamane and graphenic domains, so random stacking is likely to occur within the C—C interlayer in diamane.^[^ ^36^, ^51^ ^]^ Moreover, when pressure is used in the synthesis, inhibition of graphene is reversible but this property is absent in single‐layered graphene.^[^ ^50^ ^]^

#### 3.1.2 Electronic Properties

**Figure 4:**
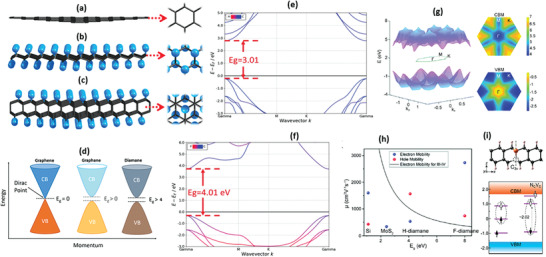
a–c) Atomic structures of the atomically thin carbon films (graphene‐ single layered graphane ‐bilayered diamane with (111) stacking surface). Reproduced with permission.^[^ ^61^ ^]^ Copyright 2021, American Chemical Society. d) Schematic showing the Dirac points of graphene, graphane, and diamane. Graph of experimentally calculated bandgaps of e) hydrogenated and f) fluorinated diamane. Reproduced with permission.^[^ ^69^ ^]^ Copyright 2021, Royal Society of Chemistry. g) 3D band structure and the band projection in the first Brillouin zone for the conduction band minimum (CBM) and valence band maximum (VBM) of a bilayered fluorinated‐diamane. h) The correlation between carrier mobility *µ* and bandgap *E*_g_ for FD and HD, with bulk III–IV semiconductor compounds as comparisons. The calculated data for electron/hole mobility are plotted in red/blue symbols. g,h) Reproduced with permission.^[^ ^70^ ^]^ Copyright 2020, Royal Society of Chemistry. i) crystal structure of hydrogenated‐diamane with an NV defect along with the electron mobility graph. Herein, the majority and minority spin states of defects are depicted as ↑ and ↓ arrows. The states above Fermi level are unoccupied, shown by unfilled arrows, and the two‐level systems are marked with dashed ellipses. Reproduced with permission.^[^ ^42^ ^]^ Copyright 2019, American Chemical Society.

Electronic properties of molecular systems depend on the availability of free electrons and their conduction. Diamane does not depress the scientific community with its electronic properties owing to the inimitable electronic structure and fundamental hexagonal carbon skeleton units (**Figure** **4**a‐graphene, 4b‐single layer graphane, and 4c‐bilayer diamane with (111) surface). The utmost graphene layer presented in diamane offers opportunities for tuning the electrons throughout the molecular system by functionalization. Graphane, diamane, and graphene, sharing identical graphitic skeleton and identical repeat unit (hexagonal carbon), exhibit similar energy band structures, direct band structures, and electronic properties. However, they have different band gaps and unique Dirac points (“the linear intersection of two bands at a single point in momentum space”), as schematically presented in Figure 4d.^[^ ^31^ ^]^ Electronic properties and band gap of graphene can be enhanced by various techniques, such as the destruction of the bond network through functionalization, doping of heteroatoms, forming diamanes, converting graphene into graphene ribbons, and even by breaking the sub‐lattice symmetry and periodic potential.^[^ ^62^, ^63^ ^]^ Though diamane showed a better band gap (Figure 4d) than graphene (resembles as semi‐metal), its electronic behavior can be further enhanced/tuned using pressure, substituent, and also through the manipulation of Brillouin zone during the hydrogenation/functionalization. The electronic band gap manipulation is very easy in the case of diamane than in diamond, graphite, and graphene.^[^ ^55^, ^61^ ^]^ For example, Li et al. tried to explore the physical properties of diamanes anchored with different anion groups using DFT calculations.^[^ ^55^ ^]^ They reported that 12 stable conformers were produced based on many unstable ones.^[^ ^51^, ^55^, ^56^, ^57^, ^64^, ^65^ ^]^ These 12 conformers exhibited direct semiconductor behavior with bandgaps fluctuating from 2.527 to 4.153 eV, and the in‐plane stiffness was greater than that of graphene.^[^ ^61^ ^]^ A key investigation of Leenaerts et al. regarding the bandgap manipulation in hydrogenated and fluorinated diamane is presented in Figure 4e,f.^[^ ^64^ ^]^ Similarly, looking at the stacking configuration for electrical properties, we realize that the formation energy is the lowest and the bandgap is the widest in the Bernal stacking of graphene. However, when the number of layers increases, the bandgap and quantum confinement effect decreases.^[^ ^48^ ^]^ After the increase in layers, the direct bandgap is converted to an indirect bandgap, which greatly influences the electron carrier mobility and quantum tunneling.^[^ ^50^ ^]^ Recent theoretical updates indicate that the nature of surface manipulation and film thickness, both influence the location of the valence band at the top of the graphene layer and the conduction band at the bottom of the graphene layer.^[^ ^34^, ^59^, ^66^ ^]^ This specific change could be utilized to incorporate desired semiconducting properties into diamane.^[^ ^51^ ^]^ Such phenomenon makes diamane an ideal candidate (unique conductivity, insulation, and ferromagnitivity) for applications in nano‐electrical, nano‐sensors, and quantum devices.^[^ ^67^, ^68^ ^]^

Pristine diamane with appropriate hydrogen to carbon ratio possesses a narrow bandgap and is an insulator in nature, but the bandgap can be modified.^[^ ^1^, ^51^ ^]^ In contrast, using Bernal stacked bilayered graphene during functionalization to open the bandgap, by breaking the inversion symmetry and tuning gap energy, has provided the possibility for its use as a semiconductor (as an alternative to silicon). The negative effects of dehydrogenation on the electronic properties of diamanes have also been observed in the case of lattice distortion beyond a certain limit.^[^ ^50^ ^]^ To comprehend the uniqueness of band structure (undeniably, the band structure of the diamane films requires further investigation to fully understand, but many studies have approved a bandgap as accessible in Figure 4g) of hydrogen and fluorine in diamane, external electrical field and band projection is influential. Diamane consisting of these atoms and its band projection in the first Brillouin zone for the conduction band minimum and valence band maximum in case of bilayered F‐diamane (Figure 4g) specifies the transition of the direct‐indirect bandgap for films at a definite thickness. This process can be further modified by using external electric fields. Similarly, perpendicular electric field can also tune the bandgap of diamane because the electric field monotonically decreases the bandgap of bilayered diamane thus, modifying it's electronic behavior.^[^ ^34^, ^59^, ^66^ ^]^ Therefore, electric field is an effective means applied to alter the electronic characteristics of diamane by causing defects in diamane (hole and vacancies) which can be used for the generation of super‐lattices.^[^ ^68^ ^]^ Similarly, according to Pavel et al., the reduced screening in diamanes and their derivatives hasten the radiative electronic transition rate up to 1 GHz for the 2.02 eV transition in diamane (Figure 4h), versus 97 MHz for the 1.42 eV transition in the diamond nitrogen‐vacancy center. A smaller Bohr radius and higher radiative rate in the diamane host make it further superior to graphane, diamond, and other similar systems for single‐photon emission.^[^ ^61^, ^68^, ^71^ ^]^ A recent study carried out by Liangbing et al.^[^ ^64^ ^]^ has shown that the band of diamanes functionalized with fluorine is higher than that functionalized with hydrogen, as shown in Figure 4e,f. Similar passivation performed to bilayered graphene opens a direct bandgap, however if passivation is neglected then the semiconducting state is switched back to a semi‐metallic state.^[^ ^46^, ^71^ ^]^ In the case of hydrogenation, the electronic properties of hydrogenated diamane principally are determined by the arrangement and concentration of hydrogen atoms adsorbed at the sites of the carbon skeleton, as discussed previously and shown in Figure 4e,f.^[^ ^51^, ^72^ ^]^ In addition, the type of functionalization/dopant in the graphane layer of diamane greatly affects the electron mobility and hole mobility as depicted in Figure 4h,i).^[^ ^50^, ^51^, ^72^ ^]^ In this regard, Li et al. reported the modification of ultra‐thin hydrogenated diamond (UTHD) by substituting the carbon atoms with lithium and phosphorous, and confirmed the electron distribution in the n‐type doped semiconducting material.^[^ ^55^ ^]^

Different functionalization strategies bring in new functionalities. For example, fluorination can modify the electronic properties of diamane (if it is functionalized from both sides, the fluorinated diamane will be insulator; however, if it is functionalized from only one side it will be conductive). Semi‐fluorinated diamanes with one side conducting and the other side insulating are termed as Janus F‐diamane, which become a hot topic for theoretical and computational studies.^[^ ^73^, ^74^ ^]^ The high electronegativity of fluorine atoms provides significant charge transfer in fluorinated configurations, affecting the electron density on the surface. These conditions will be reflected in the interaction and compatibility of diamane with other media. Thus, modification of band gap for semi‐conduction applications can be achieved by the incorporation of fluorine atom to obtain new physical characteristics.^[^ ^71^, ^75^, ^76^ ^]^ Fermi velocity of semi‐metallic state could either increase or decrease the band gap, depending on the nature of the interlayer bonded domain (AA or AB) during fluorination. Moreover, fluorination also led to new structural features, altered symmetry, and electronic structures, which allows for the precise tuning of sp^3^ hybridized carbon in diamane.^[^ ^76^, ^77^ ^]^ Using silicon dopant or creating silicon‐vacancy has also been reported to widen the bandgap in diamane, especially for quantum mechanical studies.^[^ ^77^ ^]^ Meanwhile, the use of pressure and interlayer hybridization has also been demonstrated to tune the electronic structure of the pristine graphene layer presented in diamane.^[^ ^66^ ^]^

Electronic properties of diamane directly depend on the nature of graphene (starting materials) used for the fabrication of diamane. This has been demonstrated by using twisted bilayered graphene (TBG) to fabricate diamanes, which resulted in diamanes with different properties from those using a normal bilayered graphene. In a normal bilayered graphene, that is, when the twist angle is zero in an AB stacked bilayered graphene, its bandgap also remains zero along with energy‐momentum dispersion which is quadratic in nature.^[^ ^69^ ^]^ On the contrary, TBG forms the Moire's pattern (Figure 3g), which creates ordered superlattices of finite diamond‐like crystals in the graphene layers, depending on the twist angle. Therefore, the enhancement of bandgap at a certain degree of twist angle is possible. This resulted in the superposition of rotated layers.^[^ ^69^ ^]^ Hydrogenation of TBG produces diamane with Moire's pattern dissimilar to that of graphene and normal diamane. These changes in electronic structure influence the electronic spectra, frequency shifts, presence of active phonon modes, and quantum peculiarities of hydrogenated diamanes.^[^ ^50^, ^70^, ^78^ ^]^ During the functionalization or substitution, not only the band gap is altered, but the superconductivity, insulation, and other important electronic properties are also affected. The interlayer interaction of bilayered graphene provides the characteristics of superconductivity in its magic‐angle of 1.1˚.^[^ ^48^ ^]^ The coupling of electronic phonon will also help to provide superconductivity in diamane which is useful for the next generation of nanoelectronics.^[^ ^60^ ^]^

**Figure 5:**
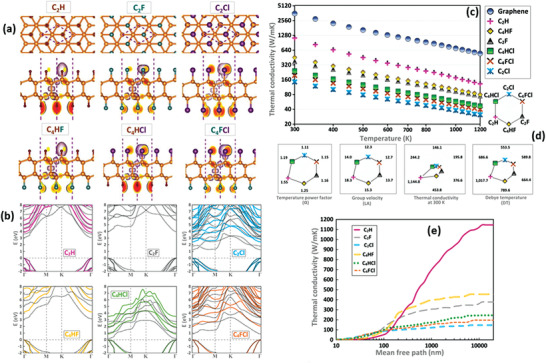
a) Atomic structural units of diamane nano‐sheets used by Raeisi et al. to study the nature of Janus and non‐Janus structured diamanes. Herein, the iso‐surfaces (set at 0.7) demonstrate the electron localization function inside the unit‐cell. b) PBEsol (dotted lines) and hybrid HSE06 (continuous lines) approaches based on theoretically calculated band structures of diamane monolayer (with Janus and non‐Janus structures). In this work, valance band maximum was set to zero energy. c,d) Lattice thermal conductivities of Janus and non‐Janus diamanes which is calculated based on theoretical parameters as a function of temperature. The lower panels (d) in the graph exemplify the temperature power factor, the group velocity of in‐plane longitudinal (km s^−1^), thermal conductivity at room temperature (W mK^−1^), and Debye temperature (K), correspondingly. e) Cumulative thermal conductivity of diamane nanosheets (Janus and non‐Janus) as a function of mean free path. Reproduced with permission.^[^ ^59^ ^]^ Copyright 2020, Elsevier.

Another class of diamanes called Janus and non‐Janus structured diamanes, exhibits exceptional thermal and electrical conductivity and quite different physical properties in comparison to normal bilayered diamanes. Recent theoretical results reported by Raeisi et al. are summarized in **Figure** **5**a–e, which can be used to guide the fabrication of new hybrid materials using diamanes and their derivatives. These diamanes are also promising semiconducting materials for the construction of next generation nanodevices.^[^ ^61^ ^]^ The work carried out by Raeisi et al. highlights the considerable role of different functional moieties in the thermal and electronic conduction responses in the diamane nanosystems.^[^ ^59^ ^]^ In his work, Raeisi et al. used first‐principles density functional theory simulations by employing the Vienna Ab‐initio Simulation Package.^[^ ^59^ ^]^ The structures showing (Figure 5b) dual properties known by Janus structures exhibit different bandgap characteristics in comparison to the normal diamane and graphene. The electronic and thermal conduction of Janus diamanes falls in between their non‐Janus counterparts and twisted diamane.^[^ ^59^ ^]^ Henceforth, the effect of functionalization on the electronic band structure and the huge potential of novel electronic properties of diamane inspired the exploration for new 2D materials with interesting properties. The fact that diamane can be converted to graphene after the removal of functional groups has also made them unique.

#### 3.1.3 Magnetic Properties

The magnetic behavior of diamane and its derivatives is very interesting which opens up new theoretical and experimental research fields with immense potentials. Magnetic behavior normally emerges when diamane is substituted or doped with atoms rather than performing functionalization. The magnetism of diamane is close to the graphene doped with boron, nitrogen, sulfur, or other suitable dopants for the creation of vacancy with different charged states. Similar to graphene, these dopants close the energy gap, narrow the bandgap, and open magnetic properties rather than electronic properties of diamane.^[^ ^41^, ^54^ ^]^ Strong ferromagnetic and paramagnetic behavior have been demonstrated in diamanes in the case of nitrogen doping, owing to the involvement of lone pair electrons of nitrogen in ring current. However, nitrogen substitution in the case of graphene showed weak ferromagnetic behavior, while nitrogen‐vacancy on the surface showed paramagnetic performance, owing to more defects on edges of graphitic carbon.^[^ ^51^, ^58^, ^59^, ^60^, ^61^ ^]^ Captivatingly, ferromagnetic behavior becomes dominant at a high concentration of holes in nitrogen substituted diamane, exhibiting new magnetic behavior in quasi‐particles.^[^ ^33^, ^39^, ^40^, ^41^, ^42^ ^]^ Creating silicon (Si) vacancy also slightly enhances the paramagnetism and spin ordering state in diamane. As reported, lowering the Bohr radius and increasing the radiative rate make diamane different from normal non‐doped diamanes in single‐photon emission. This phenomenon in turn affects its magnetic properties.^[^ ^42^, ^77^ ^]^ The application of electric field or desorption of hydrogen from the surface of graphene also decreases the bandgap of diamane, suggesting a 2D ferromagnetic semiconductor with spin‐polarized bands.^[^ ^50^, ^56^, ^57^, ^58^, ^59^, ^60^, ^61^ ^]^ Semi hydrogenation promotes an indirect small bandgap different from graphene and showcases ferromagnetism while coupled with magnetic moments. An energy gap from small to zero is seen when an electric field is applied.^[^ ^50^, ^75^ ^]^ The outermost three‐layer of un‐hydrogenated sides in semi‐hydrogenated structures of diamond films have also shown ferromagnetic properties.^[^ ^79^ ^]^ In the case of hydroxylated diamanes, they showed insulative behavior for ferromagnetism.^[^ ^48^ ^]^ Thus, diamane exhibits magnetic properties relating to both conductive and insulative behavior. These properties are unique among other 2D nanostructures and thus, could play a crucial role in the electromagnetism sector of nanotechnology.

#### 3.1.4 Optical Properties

Along with electronic and magnetic properties, diamanes show exceptional optical properties. The optical properties of diamanes and their derivatives are still not clear and further investigation is needed.^[^ ^45^, ^49^, ^80^, ^81^ ^]^ Based on the available literature, the wide bandgap and direct energy gap in diamane are responsible for its optical properties, which can be altered by using several external factors.^[^ ^40^, ^41^, ^42^, ^43^, ^44^, ^45^ ^]^ Flexible and transparent materials of functionalized diamane have been fabricated which could be useful in nano‐photonics devices.^[^ ^1^, ^80^, ^81^ ^]^ The optical peculiarities, wide‐gap spectra, and many resonance peaks in valent and conductive bands in the density of states are seen as enhanced optical properties in diamanes, which are required for the smooth operation in various optical applications.^[^ ^50^ ^]^ Correspondingly, the functional moieties of fluorine, chlorine, and hydrogen when added to the bilayered graphene also enable enhanced optical properties in diamanes.^[^ ^72^, ^73^, ^74^ ^]^ It is reported that chlorinated diamanes absorbed light over a wide energy range in the visible regions, whilst fluorinated and hydrogenated diamanes are incapable of absorbing visible light, because of the drastic change in electronic structure.^[^ ^80^ ^]^ Meanwhile, fluorinated diamane which is an insulating diamond layer has the capability of performing electron beam irradiation. This property can be used in nano‐patterning techniques, and the situation of wide bandgap and direct energy gap provides a base for optical applications.^[^ ^35^, ^82^ ^]^ The silicon‐vacancy in diamane provides the single‐photon emission at near‐visible spectra with narrow bandwidth. These photon sources providing photoluminescence emitting from single‐photon sources could offer revised optical properties.^[^ ^77^ ^]^ Heterostructures of hydrogenated graphene and TBG forming Moire's diamanes, could narrow the mini bands inside ultra‐wide 2D bandgap, therefore enhancing the optical properties of the structure.^[^ ^47^ ^]^ The substitution of atoms instead of functionalization in UTHD films tunes the optical properties too, due to dielectrics and wide bandgap.^[^ ^55^, ^83^ ^]^ Thus, various functional groups, either via substitution or via functionalization, could bring in exceptional optical properties to the unique diamanes.

#### 3.1.5 Thermal Properties

Thermal conductivity is influenced by heat transport which ultimately, is influenced by acoustic mode and optic mode. The acoustic mode should be dominant and overlapped to provide high thermal conductivity.^[^ ^84^ ^]^ Along with heat transport, group velocities also influence thermal conductivity. An increase in group velocity increases the thermal velocity and width of dedicated frequency for acoustic bands.^[^ ^59^ ^]^ Therefore, the acoustic mode relating to out‐of‐plane (ZA) phonon and stacking order allows for giant thermal conductivity even at room temperature. Thus, high thermal conductivity can be achieved when the low frequency of optical mode forms increased group velocity. Graphite, pristine AB bilayer graphene (diamane), graphane, and diamond are near zero thermal expansion materials at room temperature. The incorporation of interlayer bonds can engineer the thermal conductivity of these 2D systems, as demonstrated by several excellent reports.^[^ ^70^ ^]^ In terms of the structural configurations, we note that the stacking sequence of lonsdaleite (AA) has an higher conductivity than that of Bernal (AB), due to the horizontal reflection symmetry restricting the phonon scattering channels. At different temperatures, the thermal conductivity changes, which decreases with the increase in temperature, owing to the hindrance at edge boundaries of conduction pathways.^[^ ^70^, ^85^ ^]^ Moreover, it is observed that the stability of diamane is directly dependent on the biaxial strain (about 20%) and an increase in strain reduces the thermal conductivity of diamane and its derivatives.^[^ ^86^ ^]^ The thermal mode in graphene and diamane is normally seen with ballistic thermal conductance, but the thermal conductivity of diamane can be influenced by deformation caused by increasing the number of layers of graphene or by decreasing the hydrogen content. However, this technique is not possible in graphene, as it only contains carbon atoms.^[^ ^50^ ^]^ Hence, all the factors mentioned above may directly or indirectly influence the thermal conductivity of diamanes.

Fluorination, hydrogenation, etc. have been carried out in bilayered graphene to produce diamane with improved thermal properties. Enormously high thermal conductivity has been demonstrated by diamane via hydrogenation under controlled experimental conditions.^[^ ^33^, ^84^ ^]^ Nonetheless, for high‐temperature applications, hydrogenated diamanes have high thermal conductivity and are very interesting. Particularly, the AA stacked hydrogenated diamane has greater thermal conductivity than that of the AB stacked hydrogenated diamane, due to horizontal mirror symmetry.^[^ ^84^ ^]^ Importantly, thermal transport is dominated by phonon modes which switch from ZA phonon modes in hydrogenated diamane; whereas fluorinated diamane has low‐frequency phonon modes switching to optical modesand therefore, leads to lower thermal conductivity. The intensive phonon scattering and reduced group velocity along with influential optical modes lead to reduced thermal scattering in fluorinated diamanes.^[^ ^84^ ^]^ Therefore, along with chlorinated diamanes, fluorinated diamanes are highly resistant to heat for high temperature applications, compared with hydrogenated diamanes. The AMID simulation for a period of 20 ps has confirmed the high tolerance of fluorinated and chlorinated diamanes, subjecting to heat up to 1000 K and 500 K, respectively, but hydrogenated diamanes did not show high heat resistant behavior.^[^ ^61^, ^80^ ^]^ Thus, the thermal properties of diamanes and their modified forms provide both excellent thermal conductivity and great insulation performance, depending on the functionalization process used, which consequently make them unique.

#### 3.1.6 Mechanical Properties

For any materials, mechanical strength and durability are crucial for applications. Mechanical performances including deformation, tensile strength, strain, elasticity, and vibration are the key parameters that require thorough investigations for nanodevice fabrications.^[^ ^51^, ^56^, ^58^, ^64^, ^65^ ^]^ In general, the relative size of a finite interlayer and the residual stress at the interface influence the mechanical responses, as they could change the layered carbon nanostructures from brittle to ductile.^[^ ^65^ ^]^ Interlayer bonds exhibit much stronger resistance to shear, which affects the structural integrity of the system and enhances the mechanical property.^[^ ^70^ ^]^ Diamane is harder and shows superior strength to graphene, due to the increased coefficient of stiffness, however it is more brittle than graphene and its derivatives.^[^ ^1^, ^34^, ^44^, ^48^ ^]^ It is estimated that the stretching and bending of bilayered graphene (normal diamane) are much higher than graphene which makes it a better alternative for nanotechnology applications. The functional moieties determine the ratio of sp^2^–sp^3^ hybridization state of carbon, therefore affecting the coefficient of the finite interlayer connected domain of carbon. This ultimately, influences the stiffness and other mechanical properties of diamane.^[^ ^31^, ^44^, ^61^ ^]^ When isotropic elastic properties are dominant, owing to the functionalization and disturbance in symmetry, the nano‐sheet of diamane shows the lowest elasticity and tensile strength. These characteristics are similar to those of increased thickness and lack of functional groups in graphene.^[^ ^80^ ^]^ Meanwhile, the random insertion of interlayer sp^3^ C—C nanotubes and graphene layer stacked in AA conformation enhances the interlayer shear strength, resulting in the uniform load transfers between layers, thus improving the buckling resistance. However, this reduces the Young's modulus and tensile strength as a tradeoff.^[^ ^68^ ^]^ Mostly, hexagonal conformations are responsible for the exceptional properties of the highest longitudinal stiffness. High elastic moduli of diamanes are highly desirable, as they show good stiffness with great flexibility when bending.^[^ ^87^ ^]^ The addition of selected functional groups leads to different mechanical stability. For example, the incorporation of H and F increases the mechanical stability; whereas the incorporation of Cl and other big atoms reduces them. This has been verified in fluorinated diamanes that are more elastic than that of un‐functionalized diamanes.^[^ ^87^ ^]^ With increased number of sp^3^ hybridized carbon atoms in the functionalized diamanes, the tensile strength having enough flexibility will be improved for applications.^[^ ^61^, ^68^ ^]^ Functional groups on the surface of diamane directly affect the effective mass of carrier (electrons and holes) mobility, elastic modulus, and deformation potential constant, as shown by the restricted electron mobility for fluorinated diamane and hole mobility in hydrogenated diamane.^[^ ^61^, ^70^ ^]^

According to a recent report, a very small effect has been observed on the bandgaps of hydrogenated diamane and lonsdaleite diamane.^[^ ^71^, ^76^ ^]^ Diamane nano‐ribbons, similar to graphene nano‐ribbons with a width of less than 100 nm, showed excellent vibrational characteristics toward high natural frequency, low energy dissipation, and high value of *Q* factor.^[^ ^88^ ^]^ In both diamane and diamane nano‐ribbons, an increase in lattice spacing exponentially increases the vibrational frequency and decreases the stiffness of layer.^[^ ^51^, ^70^ ^]^ Mechanical properties of TBG can also be tuned via functionalization of the symmetry, and during which the periodicity of diamane must be retained along with the Moire's pattern. Moreover, the increased density of interlayer bonds also improves the shear modulus, leading to tensile strength and Young's modulus being intact and unaffected.^[^ ^68^ ^]^ Thus, diamane provides exceptional mechanical and vibrational properties of high stiffness, elasticity, vibrational frequency, and flexibility that is mandatory in nanotechnology applications.

## 4 Methods for the Synthesis of Diamanes

**Figure 6:**
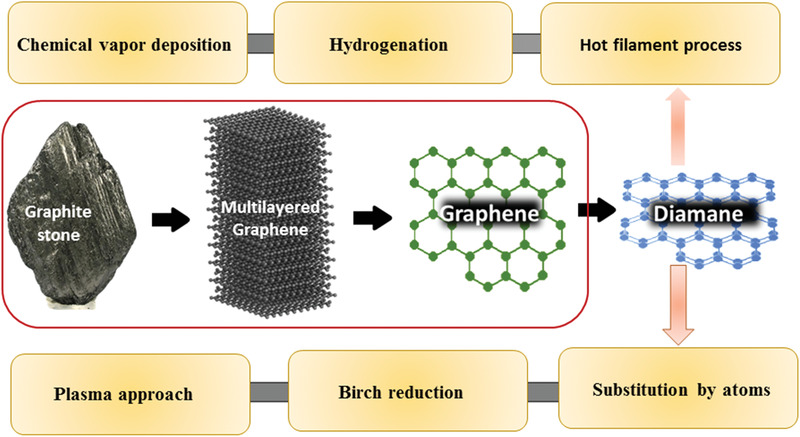
Block diagram summarizing the key methods used for the fabrication of diamanes.

Just 5 years ago, there were only a few routes for the fabrication of diamane, however several physical and chemical routes have now been reported.^[^ ^21^, ^80^, ^83^, ^84^, ^85^, ^86^ ^]^ Similar to other low‐dimensional nanostructures, the top–down and the bottom–up approaches have been utilized for the synthesis of diamane and its derivatives. The top–down approach involves the breaking down of a bulk system to gain insight into its compositional sub‐systems. Mechanical and liquid exfoliation is the most common example of the top–down approach. These methods are frequently used for the production of graphene, boron nitride, and MX phase nanomaterials,^[^ ^21^, ^80^, ^83^, ^84^, ^85^, ^86^ ^]^ and they could combine atomic or molecular units together for the creation of complex systems. The CVD and atomic layer deposition are the most common examples of the bottom–up approach and are widely used for the synthesis of 2D nanostructures with desired thickness and properties.^[^ ^81^, ^89^, ^90^ ^]^ In recent years, bottom–up routes have been used for the synthesis of diamond and nano‐diamonds which are very crucial for the research of diamond quantum wells.^[^ ^83^, ^84^, ^85^, ^86^ ^]^ For the fabrication of diamane, top–down methods are very convenient and cost‐effective for small amounts of samples. Several routes are summarized in **Figure** **6**.^[^ ^21^, ^80^, ^83^, ^84^, ^85^, ^86^ ^]^ Their detailed advantages and disadvantages will be discussed later.

### 4.1 Method of Synthesis

Graphite and graphene are the primary precursors for the synthesis of diamane. In general, pure bilayered graphene (consisting of only carbon atoms) was first produced via a top–down or bottom–up approach, and then functionalization was carried out using different chemical moieties. During the synthesis, various challenges need to overcome to achieve high‐quality diamanes, which include the selection of precursors, effects of substrates, formation of wrinkles, management of van der Waal interactions, growth kinetics of the materials, and scale‐up process.^[^ ^86^, ^87^ ^]^

To assist experimental processing with good quality diamanes, various computational tools (Gaussian, DFT, OctaDist, CADPAC, quantum chemistry program, GAMESS, Comsol Multiphysics, etc.) have also been utilized, helping to understand the stability, physical, chemical, and mechanical properties along with the growth mechanism.^[^ ^21^, ^61^, ^80^ ^]^ Computational studies played a vital role in the development of fundamental theoretical aspects (mainly structural, electronic, mechanical, thermodynamic, and quantum mechanical properties) of new materials, and assured high‐quality experimental work.^[^ ^81^ ^]^ An atomistic computational model involving the first principle has been employed to assess the thermodynamic stability, kinetically stability, inter‐layer attractions, etc. in the formation of diamanes. Macroscale models of gas flows, heat transfer, mass transfer, chemical reaction, reactive empirical bond‐order (REBO)/adaptive intermolecular reactive bond order (AIREBO) potentials, and material genome approaches are used to understand the 2D nanomaterials theoretically.^[^ ^61^ ^]^ Notably, the REBO/AIREBO model has been successfully used to describe the breakage, formation, and change in the hybridization of covalent bonds of diamanes. It can predict various complications arising from the interaction between the bulk carbon of substrate and carbon atoms produced during the chemical vapor deposition process.^[^ ^81^ ^]^ This model has also been applied to analyze the internal stress and mass density. During the last 3 years, several groups have used DFT calculations to analyze the effects of pressure on hydrogenation, change in the hybridization of bilayered graphene, intrinsic properties of fully hydrogenated diamane, lonsdaleite, silicon dopant, and silicon‐vacancy defects in graphene, graphane, and diamanes.^[^ ^69^, ^71^, ^75^, ^77^, ^91^, ^92^ ^]^ The martensitic nucleation under static compression for converting hexagonal graphite to diamond through the pole mechanism has been analyzed using this model.^[^ ^1^, ^93^ ^]^ Overall, the computational study could provide a general guide to understand the stability, symmetry, and formation of diamanes.^[^ ^40^, ^94^ ^]^

#### 4.1.1 CVD Approach

CVD is frequently used for the fabrication of diamane and its analogues. In this method, chemical vapors from various sources were used for the fabrication of graphene layers and consequently, diamanes. Different types of templates and catalysts (e.g., various forms of Co, Ni, Fe, and their alloys) were used for the production of diamane and other 2D nanomaterials, similar to graphene. The impact of catalyst and template on the quality, thinness, and properties of 2D materials has been comprehensively discussed.^[^ ^69^, ^70^ ^]^ In diamane, the stability of hybridized sp^3^ structures derived from the pristine sp^2^ graphene is very crucial, which can be achieved through a high‐quality CVD technique where the vapor deposited only onto the exterior surface of the bilayered graphene to form diamane. However, the edge deformation to a small degree is very common during CVD deposition, which defines the drawback of this technique.^[^ ^69^ ^]^ Chemisorption is a crucial step in CVD, as it is ultimately responsible for the stabilization of diamanes. A refined CVD process can passivate the dangling bonds in the pristine bilayered graphene, enabling the synthesis and generation of the stable embedded nanostructures, referred to as diamane.^[^ ^76^ ^]^ During this CVD process, the adsorption of functional groups would require a slight addition of pressure for a fraction of a second. If no pressure is applied, a splice in the graphene sheet can be observed.^[^ ^78^ ^]^ However, several CVD syntheses of diamane and its functionalization at atmospheric pressure have also been reported.^[^ ^62^, ^79^ ^]^ The functional groups in the chemical vapor source initiate the thermodynamic feasibility for the phase transition from graphene to diamanes.^[^ ^70^, ^95^ ^]^ Chlorine, hydroxyl, oxygen, ammonia, and water along with hydrogen and fluorine are typically the functional group sources used for the synthesis of diamanes by the CVD process. In addition to the above vapor sources, other chemical moieties such as metal contacts/metal surfaces (e.g., Pt, Ir, Ru, Cu, etc.), moderator (e.g., hydrogen, graphene/SiO_2_, cooling rate, etc.) and catalyst support (Ni and Co foils) have been investigated for the high‐quality diamane production. These CVD parameters are very similar to those used for graphene production. After the successful synthesis of plain bilayered 2D graphene by CVD, single and double‐sided functionalization could provide new thrilling physical properties for diamanes and their derivatives.^[^ ^73^ ^]^ To functionalize the bilayered graphene from both sides, the requirement for supporting substrate could be eliminated.^[^ ^78^ ^]^ However, the stacking order could be different within the diamane sheets owing to the involvement of subsequent transfer of two or more domains of single‐layered graphene during the CVD process.^[^ ^36^ ^]^ Notably, not only normal diamanes but also the Janus diamanes with heteroatoms (F, H, B, N, etc.) on the outer surface of diamane (bilayer graphene) have been fabricated by CVD.^[^ ^31^, ^59^ ^]^ Other dopants, such as Si in diamanes, has recently been demonstrated to enhance the electrical, magnetic, and optical properties through the CVD process.^[^ ^77^ ^]^ To control the thinness and defect of metal contact, catalysts are the driving factor for diamane formation.^[^ ^62^, ^96^ ^]^ The supporting structure of metallic parts can be applied to the bilayered graphene, providing an extra assistance for the general vapor to promote the synthesis of diamane. Cu/Ni foils enabling the AB stacking in graphene offer the basic building blocks for the formation of diamane.^[^ ^90^ ^]^

Suitable template, nature of catalyst, temperature, and pressure are required for the CVD fabrication of diamanes. These experimental conditions play a crucial role in the structural properties of diamanes. If functional groups are limited to the surface of diamanes, defects can exist at the nucleation barriers whose height determines the maximum growth of functional groups chemisorbed onto the layers of diamanes.^[^ ^62^ ^]^ The most common technique for functionalization during CVD is through fluorination and hydrogenation, which is discussed below:

#### 4.1.2 Fluorination

**Figure 7:**
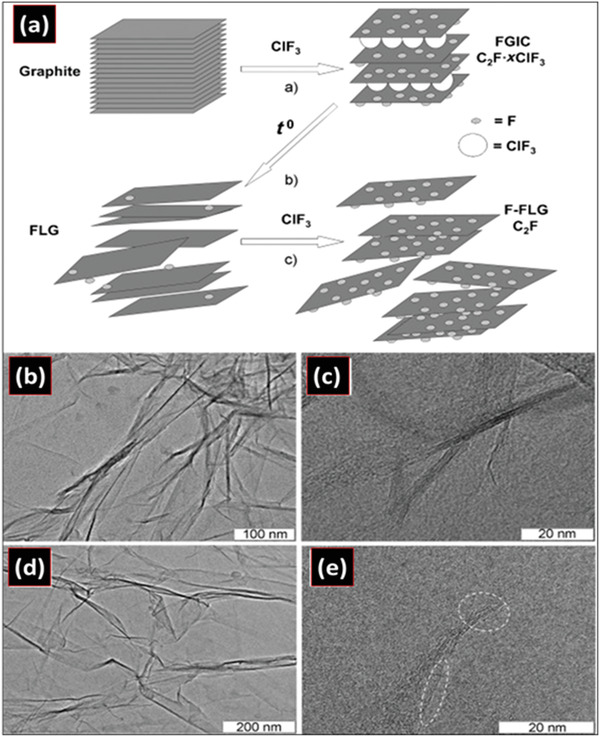
a) An illustration of the synthesis steps of few‐layered fluorinated graphene and related materials at room temperature (using fluorinated graphite), as described by Grayfer et al.^[^ ^49^ ^]^ TEM images of b,c) graphene before fluroination (3–5 layers), and d,e) fluorinated diamane. a‐e) Reproduced with permission.^[^ ^49^ ^]^ Copyright 2013, Wiley.

**Figure 8:**
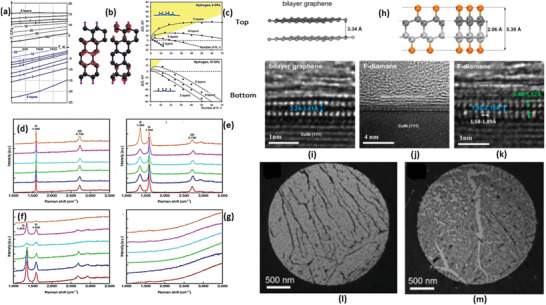
a) The phase diagram of different layered graphene to diamond film with the pristine (black) or fully hydrogenated (blue) surface. Reproduced with permission.^[^ ^101^ ^]^ Copyright 2014, American Chemical Society. b) Iso‐surface of charge densities for the valence band maximum (VBM) and conduction band minimum (CBM) for a pressure‐derived, five‐layered, hydrogenated diamane. Reproduced with permission.^[^ ^91^ ^]^ Copyright 2011, IOP Publishing Ltd. c) Gibbs free energy Δ*G*(*n*) as a function of the number *n* of H atoms chemisorbed on the graphene surface, without external pressure (top) and at 10 GPa (bottom), exemplifying the increase of the nucleation barrier for diamane creation in graphene with more than two layers. Reproduced with permission.^[^ ^62^ ^]^ Copyright 2020, Wiley‐VCH. Raman characterization of the fluorinated BLG on the CuNi (111) surface. d–g) Raman spectra at six randomly chosen positions by 488 nm excitation of the as‐grown BLG (a), Sample c, 2–3 h fluorination; (b), Sample b, ≈6 h fluorination; (c) Sample a, >12 h fluorination. d‐g) Reproduced with permission.^[^ ^35^ ^]^ Copyright 2019, Springer, Nature. h) Top: Optimized models of the bilayered graphene and F‐diamane. Orange and grey spheres represent the fluorine and carbon atoms, respectively. Bottom: Cross‐sectional TEM images of the as‐grown bilayered graphene and F‐diamane with the highlighted interlayer and interatomic distances. Reproduced with permission.^[^ ^35^ ^]^ Copyright 2019, Springer Nature. (i–k) TEM images of the two layered graphene films and l) before and m) after the hydrogenation (i.e., fabrication of diamane using pure graphene). i–k) Reproduced with permission.^[^ ^35^ ^]^ Copyright 2019, Springer Nature. l‐m) Reproduced with permission.^[^ ^102^ ^]^ Copyright 2020, Elsevier.

The process of fluorination provides the desired product by reacting materials with fluorine‐containing compounds. The stability of fluorination is higher than hydrogenation at ambient conditions.^[^ ^48^ ^]^ The important quality of fluorination includes the hydrophobicity which arises from the high electronegativity of fluorine. The electronegativity of fluorine changes the surface chemistry of the compound to be hydrophobic. Thus, after carbon‐fluorine bonding and micro‐texturing, super‐hydrophobicity can be realized. Fluorination of graphite occurs at high temperatures. Fluorination may also provide characteristics similar to Teflon in graphene which will be useful in various applications.^[^ ^97^ ^]^ The reactivity toward fluorinating agents increases when the carbon lattice shows curvature and the chemical modifications stabilize the 2D structure. The energy involved in the fluorination conversion is low compared with the free‐standing layer, due to the consequence of strong and short bonds. This energy results in a low coefficient of friction between layers of exfoliated fluorinated diamane under shear. Intercalation compounds of graphene can be formed when fluorine radicals are deposited in graphite. Subsequent separation techniques are then employed to obtain fluorinated diamanes, such as to the pressure‐derived, five‐layered, hydrogenated diamane (Figure 8a–c). The fluorination reaction is affected by the higher graphitization degree, higher fluorination temperature, and high reactivity toward fluorinating agents, such as fluoride in xenon fluoride (XeF_2_), boron trifluoride (BF_3_), iodine pentafluoride (IF_5_), chlorine trifluoride (CIF_3_), tetrafluoromethane (CF_4_), dinitrogen difluoride (N_2_F_2_), etc.^[^ ^87^, ^92^, ^98^ ^]^ The above compounds have been used in the fluorination of graphene in the form of plasma. Plasma of CF_4_, XeF_2_, and CF_6_ were used to fabricate fluorinated graphene by depositing them in a pre‐prepared graphene to obtain fluorinated diamane with interlayer bonds.^[^ ^48^, ^49^, ^99^ ^]^ Using XeF_2_ at room temperature and low atmospheric pressure to bilayered graphene, fluorination is prominent. XeF_2_ has the capability to fluorinate effectively without etching the graphene, so that efficient functionalization can be realized. XeF_2_ chemisorption on the surface alters the optical and electrical properties by minimizing the conducting charges in the *π* orbitals where the optical transparency and insulating nature of functionalized graphene is observed after the action of XeF_2_.^[^ ^100^ ^]^ This method leads to the sequential fluorination of the top and bottom layers of the bilayered graphene^[^ ^35^, ^36^, ^80^ ^]^ Grayfer et al. have recently developed a facile route (as shown in **Figure** **7**a) for the production of few‐layered fluorinated graphene at room temperature using gaseous ClF_3_ as the fluorine source. Their end product can be a good precursor for further fabrication of diamanes, diamanoids, and related materials.^[^ ^49^ ^]^ TEM images of the few layered graphene and the fluorinated graphene are presented in Figure 7b–e. TEM images showed clearly that the final product entailed of thin transparent 2D layers with no more than ten fluorographene layers stacked to each other.^[^ ^49^ ^]^ Bilayered graphene can also be fluorinated by CVD in silicon oxide (SiO_2_) where hBN also presents as the substrate to tailor its interaction with fluorine and graphene. The fluorination was realized using XeF_2_ gas which penetrated inside the graphene simultaneously, while the penetration was tuned by using rough SiO_2_ and smooth hBN. The XeF_2_ gas etched off the top hBN while the bottom hBN was protected, leaving the graphene to be fluorinated. Deposition always occurs on the top layer, so that other layers remain pristine. Hence, by tuning the penetration, the process allows for the freedom of choosing the fluorination to be done whether on the top or on the bottom surface. Single‐sided fluorination with C_4_F can open the band gaps, even at low coverage.^[^ ^100^ ^]^ For double fluorination, both the top and bottom layers of graphene are made to contact with fluorine. In another report, using XeF_2_ in a CVD process on a bilayered graphene formed the fluorinated diamane through chemisorption over a large area in crystal Cu/Ni (111) at atmospheric pressure.^[^ ^16^ ^]^ The Raman spectra of the fluorinated bilayered graphene on the CuNi (111) surface at different stages of fluorination are shown in **Figure** **8**d–h. In another method, graphite flakes were heated with fluorine gas causing fluorination to obtain (C_2_F)*_n_* materials in a large area, which allows for direct mechanical delamination of a single layered fluorinated diamane using a sticky tape.^[^ ^66^ ^]^ The fluorination process is reversible, as irradiation by an electron beam can reduce the amount of fluorine and convert it back to the AB stacked bilayered graphene.^[^ ^12^ ^]^

#### 4.1.3 Hydrogenation

Hydrogenation is a cost‐effective method in comparison to the high pressure and high‐temperature technique.^[^ ^103^ ^]^ Normally, hydrogenation refers to the process where hydrogen is reacted to certain materials to obtain desired products. Reactions can be reversible, irreversible, adsorption, and so on. The adsorption of hydrogen leads to the hybridization which changes the sp^2^ to sp^3^ propagating through the graphene layers by forming interlayer bonds. During the hydrogenation, hydrogen atoms create bonds with carbon atoms at the upper layer of sublattice A, while the lower carbon layer of sublattice A will bond with the upper carbon layer of sublattice B. The lower carbon layer of sublattice B then bonds with hydrogen atoms creating the sp^3^ hybridized state.^[^ ^58^ ^]^ Hydrogenation of bilayered graphene to synthesize hydrogenated diamane has been successful in creating thin films at the molecular level at a broad range of temperatures and pressures.^[^ ^67^ ^]^ TEM images of two layers of graphene before and after the hydrogenation were synthesized by Piazza et al, as shown in Figure 8i–m.^[^ ^35^, ^102^ ^]^ Hydrogen atoms are adsorbed on the outer layer of the multi‐layered graphite which depletes electrons to form the sp^3^ hybridization and the absence of electrons leads to the formation of bilayered graphene, whilst the hydrogen atoms stabilize the films forming the diamane.^[^ ^44^ ^]^ Hydrogenation can induce lattice changes in graphene which leads to the synthesis of hydrogenated diamane, however controlling the coverage and defects is difficult.^[^ ^35^, ^62^ ^]^ Full hydrogenation is possible, but it requires further modification to stabilize the structure. Because hydrogenation is a layer‐dependent process, controlled by hydrogenation energy barriers,^[^ ^104^ ^]^ the barrier mechanism would determine the amount of hydrogen coverage, which is associated with the processing parameters, such as the saturation rate of plasma power and exposure time. The swollen areas of the bilayered graphene adsorb hydrogen at low coverage to form nanopatterns of diamanes. Induced desorption of hydrogen to initiate electrical and chemical functionalization can be realized by annealing, resulting in dehydrogenation. Therefore, this reversible process can obtain both diamane and bilayered graphene.^[^ ^101^ ^]^ Single layered hydrogenation forms graphane with carbon bonded to one hydrogen atom above and below the layer; two‐layered hydrogenation of graphene connects carbon atoms from top layers with hydrogen and bottom layers with carbon atoms of neighboring layers, therefore forming diamanes.^[^ ^37^, ^50^ ^]^

Hot hydrogen atoms or hydroxyl radicals produced by the hot filament process, high‐pressure process, or plasma process have been used for the synthesis of H‐diamane.^[^ ^35^ ^]^ For hydrogenation, atomic hydrogen can be supplied by di‐imide (N_2_H_2_), hydrazine (N_2_H_4_), water (H_2_O), etc.^[^ ^92^ ^]^ Other methods of femtosecond laser excitation of graphite, adsorption of hydrogen on few‐layer graphene, defects manipulation in chemically modified graphene can also create interlinked diamanes, whilst hot filament and plasma processes are more frequently available than other facilities.^[^ ^44^ ^]^ So, special attention will be given to the hot filament process and plasma process.

##### Hot Filament Process

The efficient production of hydrogen by the hot filament and the possibility to process materials at low substrate temperature provide efficient way for the production of diamane. Ions of high kinetic energy in the process are accelerated towards the substrate, but the graphene surface is unaffected. Compared with the plasma treatment in which accelerated ions will etch the graphene, the hot filament process avoids such bombardments which results in the damage to graphene. This makes the hot filament process advantageous over the plasma process. Chemisorption of hydrogen atoms from the dissociation of hydrogen molecules in bilayered graphene leads to the production of hydrogenated diamane inside a hot filament reactor at low temperature and pressure. The bilayered graphene was first deposited at gold Quantifoil TEM grids before the hydrogenation in a hot filament reactor. AB stacked graphene was normally used in this process. The two tungsten wires at the reactor sites perform the heating and dissociation of pure hydrogen gas for hydrogenation. These hydrogen atoms are accelerated towards the graphene layers, for the deposition and formation of diamane nanostructures. This process was performed without the need of hydrocarbon for carburization or conditioning, because carbon contamination should be avoided when the hydrocarbon is used.^[^ ^36^, ^102^ ^]^

An alternative process for the production of hydrogen gas is from hydrocarbons. Hydrocarbon mixed with hydrogen gas has been used in a hot filament process to hydrogenate bilayered graphene for diamane, using a heated Cu substrate at low temperature and pressure.^[^ ^37^, ^50^ ^]^ Few‐layered graphene have also been attempted to create hydrogenated diamane in a hot filament reactor using a platinum (Pt(111)) substrate. Double hydrogenated diamane using a similar technique has been achieved, however the layer and stacking pattern are not well controlled.^[^ ^48^ ^]^ The thermal decomposition of hydrogen molecules produces hydrogen atoms on the hot filament surfaces which are rapidly diffused into the bulk gas. Diffusion of gas can happen on walls of reactor, which slows down the recombination reaction at typical process pressures.^[^ ^36^, ^37^, ^50^, ^102^ ^]^ Diamane is the main product formed in the hot filament process, but nano‐diamonds and CNTs are also synthesized with this process.^[^ ^36^ ^]^ Thus, diamane formation by hot filament technique is efficient, and high‐quality materials could be synthesized.

##### Plasma Approach

Plasma treatment is a simple, fast, and scalable technique where a functional group is attached to the carbon lattices. Positive ions useful in functionalization are extracted from the hydrogen plasma that fuses into the graphene sites.^[^ ^99^ ^]^ This hydrogen plasma emits enough energy through reactive species to break the double‐bonded carbon atoms and to increase the C—C sp^3^ hybridization to form diamanes.^[^ ^105^ ^]^ If the exposure time, doping, resistivity, and sample position inside the chamber are controlled, functionalization tuning can be achieved easily.^[^ ^99^ ^]^ The favorable affinity of graphene toward hydrogen was also observed during the plasma process.^[^ ^62^ ^]^ Plasma irradiation techniques can be used in the patterning of graphane nano‐circuitry and synthesis of high thermal stability diamanes.^[^ ^34^ ^]^ Carbon hybridization in AA or ABC stacked graphene is easier and simpler than in AB stacked graphene when plasma is deposited. Hybridization will lead to the change of status of the neighboring carbon atoms, non‐hydrogenated carbon layers will form covalent bonds with underlying layers, once the hydrogen coverage is large and sufficient.^[^ ^49^ ^]^

The formation of cold hydrogen plasma used for hydrogenation can be performed by using hydrogen and argon mixture at low pressure. DC plasma was ignited between two aluminum electrodes forming hydrogen plasma.^[^ ^33^ ^]^ The discharge of hydrogen plasma forms diamane in a bilayered graphene, where hydrogen atoms are chemically adsorbed on both sides of the layer forming the potential barrier of hydrogenation. Both the top and bottom layer graphene experiences hybridization of sp^3^, but the hydrogenation occurs at the top of the top layer and bottom of the bottom layer only.^[^ ^1^, ^67^ ^]^ Another process for the hydrogenation of bilayered graphene on an SiO_2_/Si substrate using hydrogen plasma was reported and subsequent heating was used to synthesize diamane.^[^ ^104^ ^]^ Cold plasma will enable the interlayer bonding between carbon atoms by chemisorption of hydrogen on top and bottom of a two‐layer graphene, where conversion can also occur on metal for hydrogenation.^[^ ^36^ ^]^ The new structure, known as triamane, is synthesized by the process of natural physisorption of water (H_2_O) above and below the surface of trilayer graphene followed by subsequent irradiation in an hydrogen plasma. Secondary and backscattered electrons could also initiate the hydrogenation process by electron impacting on the fragmentation of physisorbed H_2_O.^[^ ^34^ ^]^ The formation of diamanes can also be seen when twisted bigraphene is placed in hydrogen plasma. Temperature and pressure are applied to assist the hydrogen chemisorbing on both surfaces of graphene.^[^ ^31^ ^]^ Non‐supported bilayered graphene and trilayered graphene placed in a hydrogen cold plasma have been hypothesized to form diamane, but experimentally they are yet to be proved. In the case of deuterium adsorption, the formation of diamane up to four layers on few‐layer graphene using platinum (Pt (111)) as a substrate has also been reported.^[^ ^37^, ^50^ ^]^ Using the oxidative method, exposing oxygen gas to the bilayered graphene and multi‐layered graphene creates oxidative pits on the surface. Depending on the number of layers and the temperature, the oxidative pits were formed when hydrogen plasma was inserted into hydrogenated bilayered and monolayered graphene, however they were not seen without the hydrogenation. The oxidative pit was etched away by the hydrogen plasma and graphene layers were hydrogenated by the ions simultaneously, to form diamane.^[^ ^36^, ^50^, ^97^ ^]^ Ammonia adsorption using plasma can also lead to diamond thin films.^[^ ^67^ ^]^

The different hydrogenation processes discussed above have different mechanisms, as hydrogen plasma is more energetic than other functional groups. In the first mechanism, a long exposure time for atomic hydrogen radicals is required to generate curvature induced differences in the corrugated monolayer and smooth bilayer. This mechanism is performed outside the reactor and has less energy barrier. The long exposure time induces layer‐by‐layer modifications where the top layer is modified and etched first, then the process initiates for the second layer. The second mechanism involves the graphene being immersed inside the plasma with energetic hydrogen species.^[^ ^99^ ^]^ This mechanism is developed based on the need of experiments to achieve high‐quality diamanes. In short, the plasma method is a feasible method for hydrogenation of bilayered graphene to form diamane, but attention should be paid to the plasma deposition process to avoid the graphene layers being etched away.

#### 4.1.4 Use of Pressure

**Figure 9:**
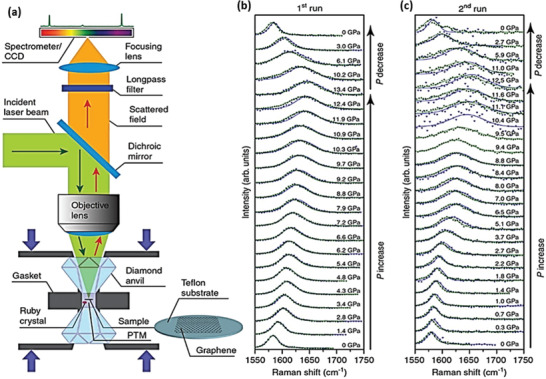
a) Schematic apparatus used for the pressure‐induced synthesis of diamane derivatives as reported by Martins et al. and b,c) the effects of pressure on the hybridization of carbon atoms presented in the diamane nanosheets evaluated by comparing the G bands using Raman spectroscopy. Reproduced with permission.^[^ ^105^ ^]^ Copyright 2017, Springer Nature.

Pressure has been used for the synthesis of diamane as an alternative to CVD.^[^ ^35^, ^105^ ^]^ This process has gained much attention from researchers, but the need for a small amount of pressure for stabilization even after the synthesis makes it difficult for applications, hence is not used frequently. Even though the two‐layered films have been fabricated without using any pressure under ambient conditions, increasing the number of layers to synthesize diamond and other related products requires the use of pressure.^[^ ^62^ ^]^ The formation of diamane using pressure can be achieved, but the stability cannot persist without external auxiliary of indentation or high pressure. The passivation by atoms or molecules to the dangling bonds is required to ensure good stability, if the pressure method is applied and stabilizing pressure is not provided. Otherwise, there is a risk of transformation from sp^3^ back to sp^2^ hybridization. Sp^2^ carbon films must be close to each other for hybridization as the interlayer distance is larger than bond length. High pressure can be used to synthesize diamane stabilized at 5 GPa.^[^ ^54^ ^]^ In the formation of hydrogenated diamane by the application of high pressure and temperature, a trilayer of graphene was compressed. Stabilization of the graphene product by providing a few GPa of pressure known as quenching must be utilized under ambient conditions after the synthesis.^[^ ^52^, ^74^ ^]^ Diamane can also be synthesized by the process of diamondization where exfoliated few layers of graphene are compressed mechanically, the energy gap is opened and dramatic resistance is increasingly changed to alter the hybridization of carbon atoms by interlayer sliding to sp^3^. Pristine hydrogenated diamane has been synthesized by applying pressure above 20 GPa in trilayered or thicker graphene up to twelve layers, but the maintenance of pressure of about 1 GPa was required for stabilization. For example, Martins et al. developed a new strategy for the fabrication of diamondene (a form of diamane) by using the high pressure technique.^[^ ^105^ ^]^ Their detailed steps involved in the process are presented in **Figure** **9**a.^[^ ^105^ ^]^ They first reported the effect of pressure on the hybridization of carbon atoms presented in the diamondene nanosheets using Raman spectroscopy. In Figure 9b,c, we can see that, as the pressure increased during the synthesis, the G band in the Raman spectra shifted toward the higher frequency. This Raman shift under pressure has been attributed to strain in the crystal lattice caused by the conversion of sp^2^ carbons to sp^3^ carbons.^[^ ^97^, ^106^ ^.^ ^107^ ^]^ Alternatively, thermal treatment at high pressure can also be used to stabilize the pristine structure and minimize the distortion in crystal lattice.^[^ ^66^ ^]^ Water can also be used to modify the graphene at high pressure, in presence of functional groups, during which the sp^2^–sp^3^ transition was observed, but when the pressure is released for a long time, recovery to sp^2^ from sp^3^ occurred, implying the metastable state of sp^3^. The presence of high pressure and hydroxyl groups is evident in producing 2D diamond‐like crystals using bilayered or few‐layered graphene films, however the process is reversible and returns to the original graphene on decreased pressure.^[^ ^46^, ^106^ ^]^ The pressure effect on bilayer graphene showed that the hardness of pressurized bilayered graphene becomes equal to diamond, and the electrical conductivity is reversibly dropped upon indentation.^[^ ^66^, ^106^ ^]^ While these abilities were decreased when the thickness of the layer of graphene was increased, the pristine few‐layered graphene could be formed through high‐pressure formation of interlayer sp^3^ C—C bond under 16 GPa pressure, whilst stabilization was mandatory (Figure 9b,c). Hexagonal pristine diamane was created with three or more layers under pressure, and phase transition pressure was decreased with several layers. The formation of pristine diamane by the use of pressure is possible, but it must be stabilized at a minimal pressure of 1 GPa.^[^ ^48^ ^]^

Thus, we can conclude that the role of pressure in the fabrication of diamane and related nanomaterials is important for the production of high‐quality diamondene, diamanes, and diamanoids, but optimization of pressure and vacuum conditions are essential. Further, this approach is extremely favored by the stacking of two or more layers of graphene having —OH and —H chemical moieties. Finally, it is a destructive process and requires an expensive instrumental setup.

#### 4.1.5 Twisted Bilayer Graphene and Birch Reduction

Graphene is stacked on top of the other in a bilayered graphene. When this stacked graphene is rotated at a certain twist angle, they are known as the twisted bilayer graphene,^[^ ^94^, ^95^ ^]^ and results in the Moire's pattern when the twisted layers are overlapped with each other. Graphene sheets are rotated at an angle “*θ*” to obtain the twisted bilayer graphene, and further its substitution/functionalization can lead to diamane. The AB stacked bilayer graphene contains a unit cell of four carbons, in which two are located as free units and the other two are positioned one above the other. Bond atoms are used for the adsorption of atoms or molecules, in contrast to diamane where adsorbed atoms alternate, as bond atoms do not combine in the process.^[^ ^47^, ^50^ ^]^ When graphene sheets was rotated at an angle “*θ*” during the fabrication of twisted bilayer graphene, creating the Van Hove singularities and flat regions near the Fermi level, exhibits superconductivity in diamane.^[^ ^78^ ^]^ The degree of freedom to control the electronic properties of bilayered graphene depends on the local alignment of the atoms in a pair of adjacent planes. The stacking sequence of AA and AB leads to cubic and hexagonal diamonds. This explains why rotating the layer from 0° to 60˚ results in different twisted bilayer graphene (diamane) with Moire patterns.^[^ ^63^, ^76^ ^]^ Similarly, twisted bilayer graphene at 30˚ has been fabricated with Moire's pattern when overlapped. Diamane was then formed after hydrogen or fluorine adsorption on both sides of bilayered graphene with a twisted angle of nearly 30˚. The bilayered graphene was first kept on the silicon dioxide (SiO_2_) via substrate interactions of hBN, and then hydrogenation or fluorination was carried out at the top and bottom of the bilayered graphene.^[^ ^47^, ^50^ ^]^

Another process for the formation of diamane is by using the Birch reduction method. Instead of hydrogen plasma, the Birch type reduction used a solution for the production of hydrogenated few‐layered graphene. In this method, lithium atom acts as a reductant and water act as the proton source, in which the efficiency depends upon the hydrogen ion (H^+^) production. Graphene flakes of single‐layered graphene and AB^−^ stacked bilayered graphene were created by the mechanical exfoliation on a silicon dioxide/silicon (SiO_2_/Si) substrate, followed with the Birch reduction to produce hydrogenated single‐layered graphene, known as graphane, and hydrogenated bilayered graphene, that is, diamane.^[^ ^43^ ^]^ In the last few years, various groups reported the production of diamane and its derivatives by chemical routes. The main drawback of the Birch reduction for the fabrication of diamane and other analog 2D systems are the purification and lattice distortion, due to the unwanted doping of lithium atoms in the carbon skeleton.

#### 4.1.6 From Graphene and Their Derivatives

Graphene is a 2D nanostructure mainly synthesized from bulk graphite. Several synthesis methods have been documented, but the most frequently used and prominent methods are the top–down approach of chemical and mechanical exfoliation of graphite, and the bottom–up approach of CVD and epitaxial growth on insulating surfaces (diamond or SiC). The bottom–up approach is hard to control the stacking order and the number of layers, but is regularly used due to its convenience. The liquid exfoliation has led to corresponding single‐layered to few‐layered platelets from bulk flakes of graphite, hBN, black phosphorus, transition metal dichalcogenides, etc.^[^ ^98^ ^]^ Exfoliation on a silicon substrate from silicon dioxide (SiO_2_) over Si wafer along with hBN by using pickup method is another method for synthesizing 2D systems like graphene.^[^ ^73^, ^99^ ^]^ The micromechanical cleavage or scotch tape technique is obviously the first technique used for graphene synthesis, which is hard to obtain monolayered structures.^[^ ^31^ ^]^ Graphene crystals have been used in the synthesis of diamanes and other 2D carbon structures by the micromechanical cleavage of graphite on top of an oxidized silicon substrate.^[^ ^33^ ^]^ Trilayered and thicker graphene have anisotropic properties, dependent on the stacking order of the graphene sheets. Trilayered graphene has been synthesized by high‐temperature annealing of n‐type Si‐terminated 6H‐SiC (0001). Mechanical milling techniques are able to increase the content of rhombohedral modifications in graphene. Exfoliating the natural graphite with rhombohedral phase mechanically using the “scotch tape method” led to graphene with ABC stacking. A modified scotch tape method for large‐area graphene flakes has also been developed, however controlling the number of layers, yield, and quality are notable drawbacks of this technique.

Graphene flakes can have different stacking orders when the scotch tape method was used, as they can make some layers twisted.^[^ ^57^ ^]^ In chemical exfoliation, graphite was intercalated by some species to increase the spacing between neighboring sheets. This process obtained graphene with uncontrollable characters. CVD was capable of fabricating monolayered and bilayered graphene, via epitaxial growth from hydrocarbon deposited on the metal surfaces. After the CVD, the graphene formed on a Cu plate was rolled on a polymer, after etching away the Cu. Thus, graphene islands were obtained.^[^ ^31^ ^]^

**Figure 10:**
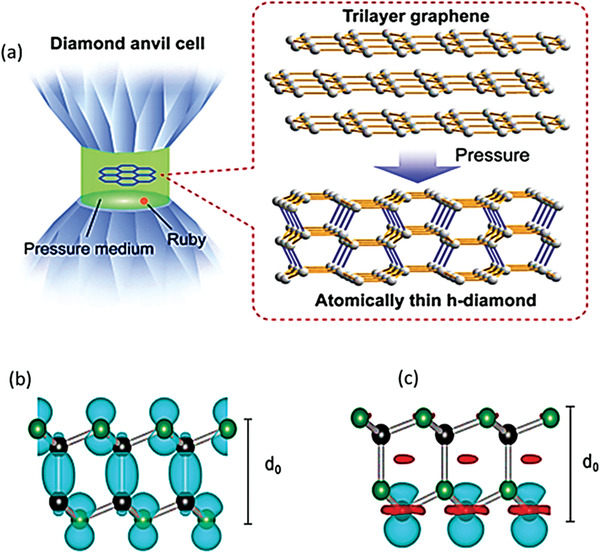
a) The synthesis of diamane through pressure application and the effect of pressure on molecular arrangement. Reprinted with permission.^[^ ^66^ ^]^ Copyright 2020, ACS. b) The relaxed structure of NCCN is presented by projecting on the [100] direction where the blue envelopes are spin up charge density of the valence band maximum at *Γ*‐point. c) The relaxed structure of CNCN is presented by projecting on the [100] direction, where blue and red envelopes are the positive and negative magnetization density, respectively, of the valence band maximum at K‐point. The black and green balls represent the C and N atoms, respectively. Reproduced with permission.^[^ ^39^ ^]^ Copyright 2020, American Physical Society.

Cu‐Ni alloy was also used in fabricating graphene using high‐pressure CVD.^[^ ^106^, ^107^ ^]^ Mechanical exfoliation using bulk graphite on Si/SiO_2_ surfaces can also produce trilayer graphene.^[^ ^66^ ^]^ The need of graphene for the synthesis of diamane and diamanoids can be fulfilled mostly by the CVD, and the introduction of pressure under suitable experimental conditions could lead to diamanes and their derivatives, as depicted in **Figure** **10**a. Thus, the existing, well‐developed graphene synthesis techniques provide a convenient base for the creation of new 2D structures, especially diamanes and diamanoids. Moreover, the procedures used for doping graphene with heteroatoms can also be applied to diamanes. Indeed, the configuration of NCCC, CNCC, NCCN, and CNCN have been reported by the substitution of nitrogen in graphene, resulting in different diamane structures, as depicted in Figure 10b,c.^[^ ^39^, ^54^ ^]^ In recent years, a variety of heteroatoms has been investigated. It is concluded that larger hetero size atoms such as sulfur caused instability and structural deformation in diamanes; whilst smaller substitution atoms such as nitrogen and boron provided additional stability.^[^ ^44^, ^63^, ^67^ ^]^

## 5 Methods for the Synthesis of Diamanoids

**Figure 11:**
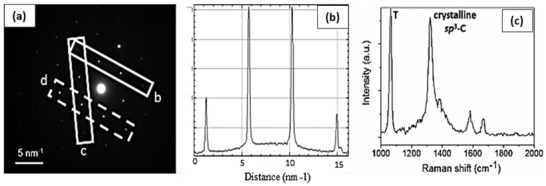
a) Typical electron diffraction pattern, b) profile plots of the diffraction peak intensities obtained from pattern region highlighted by the rectangular boxes in (a), and c) UV Raman spectrum (at 244 nm) of the FLG after the hydrogenation. Reproduced with permission.^[^ ^38^ ^]^ Copyright 2020, Elsevier.

Diamanoids are thought to be formed when stability is obtained at thicker graphene.^[^ ^94^ ^]^ It was thought that low‐quality graphene with several layers provided the basis for diamanoid formation, however the quality of original graphene and the lack of lateral resolution for observation in Raman spectra make it debatable.^[^ ^102^ ^]^ Therefore, few‐layered graphene could provide better understanding as the source materials. In a methane‐Ni substrate CVD process, the resulting few‐layer graphene was then transferred to a Cu TEM grid, using the free transfer method for minimal contamination.^[^ ^36^, ^38^ ^]^ Of course, exfoliation of graphite oxide and arc evaporation of graphite under hydrogen can be used for the formation of few‐layer graphene, as well as the chemical exfoliation and the Birch technique.^[^ ^108^ ^]^ It was found that the films were polycrystalline in an in‐plane direction and the layers were Bernal stacked (ABAB) or rotationally faulted. After the formation of few‐layered graphene, a hydrogenation process by using hydrogen and methane in a hot filament reactor induced partial or full hybridization of carbon at low pressure and low temperature, resulting in the formation of diamanoids.^[^ ^53^ ^]^ An electron diffraction pattern, profile plots of the diffraction peak intensities, and UV Raman spectrum (at 244 nm) of the resulting few‐layered graphene after the hot‐filament‐promoted hydrogenation are displayed in **Figure** **11**a. Tungsten wires were used as the heating elements which were not exposed to the hydrocarbon gas that acted as the carburization or conditioning agent, to avoid contamination. The high purity hydrogen gas was introduced from the top of the chamber, after the evacuation of the gas in front of the chamber.

The process was conducted at low temperature and pressure, dissociating the hydrogen molecules into atoms. The first layer was fully hydrogenated, similar to diamane, where half atoms were bonded with hydrogen atoms and half with carbon atoms. The second layer was not hydrogenated and was bonded with carbon atoms of the previous and other layers. The bottom of the last layer was bonded with hydrogen atoms; whilst the top was bonded with carbon atoms, leading to diamanoids. The hydrogenation in a commercial hot filament reactor with a pressure of 10 torr, using hydrogen flux, led to the formation of diamanoids.^[^ ^38^ ^]^ These synthesis techniques were performed by using the following stacking orders, ABBB, ABAB, ABCA, ABBA, and ABA, which are most likely to occur and are most stable.^[^ ^36^ ^]^ Hydrogen plasma also has the capability to hydrogenate a few layers of graphene to form diamanoids, by turning the semi‐metallic states into semi‐conducting states with a large bandgap.^[^ ^91^ ^]^ Thus, the synthesis of 2D diamonds and related materials from hot‐filament CVD is vital for the formation of diamanoids.

The thermo‐mechanical properties of diamanoids are close to those of diamanes, as primarily verified by recent theoretical studies. In this regard, Gao et al. evaluated the effect of pressure (applied by an AFM tip) on the BLG using SiO_2_ as the template at room temperature.^[^ ^109^ ^]^ They reported that the hardness was almost equivalent to that of diamond, and the electrical conductivity showed a reversible drop under the indentatio. Based on DFT calculations, they further confirmed that, upon compression, the BLG film transformed into a diamond‐like film, producing both elastic deformations and sp^2^ to sp^3^ chemical changes.^[^ ^109^ ^]^ For a comprehensive understanding, various synthesis methods and the associated properties of diamane–diamanoids, benchmarked against other 2D systems, including graphene, are summarized in **Table** **1**.

**Table 1:** Various synthesis methods and comparison of the properties of graphene, hexagonal boron nitride, molybdenum sulfide, phospherene, diamane, and diamanoids

2D Materials	Graphene	Hexagonal boron nitride^[^ ^110^, ^111^ ^]^	Molybdenum sulfide^[^ ^112^, ^113^, ^114^ ^]^	Phospherene^[^ ^115^, ^116^ ^]^	Diamane	Diamanoids
Description	Single layered allotrope of C atoms, having sp^2^ hybridization, covalently bonded.	2D crystalline analogue of the graphene, consisting of and N and B atoms (B_3_N_3_) in hexagonal.	2D metal dichalcogenide, in which one molybdenum (Mo) atom covalently bonded with two sulfide (S) atoms.	2D stable allotropic material, consisting of single or few layered black phosphorous (P).	Bilayered graphene converted from sp^2^ to sp^3^ hybridization by the attachment of functional groups on one side, and the neighboring C layers on the other side.	Few layered graphene converted from sp^2^ to sp^3^ hybridization by the attachment of H atoms on one side and the neighboring C layers on the other side.
Structure						
Structure	Covalent bonding between the C—C layers having hexagonal honeycomb lattice with three sigma (*σ*) bonds in each lattice. The edges of graphene can be classified to zig‐zag and arm‐chair conformations.	N and B atoms alternated in a honeycomb structures, and covalently bonded. The edge structure is zigzag or armchair with AA' stacking being most favorable.	Hexagonal layered structure with covalently bonded network. Hexagonal and rhombohedral symmetry with trigonal prismatic coordination.	Orthorhombic honeycomb lattice structure with covalently bonded network, with zigzag or armchair edge structure.	Bernal stacking (AB stacking) and lonsdaleite stacking (AA stacking), having hexagonal or cubic lattice. Twisted bilayer graphene also has the capability to form diamane with bond angle twisted to certain degrees.	Unstable in Bernal stacking (AB stacking), but the stacking order of (ABB), (ABBA), (ABBC), (AABBCC), diamanoids being stable. Heterostructure of diamanoids and graphene can be formed by Bernal stacking. Twisted superimposed coherent domain is also formed when one domain of diamanoids and other domain of unconverted graphenic domain are overlapped.
Properties						
Electrical properties	Exhibit (chiral) quantum Hall effects of two new kinds, with least quantum conductivity Interference effects relating to quantum suppression is resilient. A zero overlap semimetal with excellent electronic conductivity for strong electrical properties.	Large band gap and surface optical phonon modes provide excellent electrical insulation. Band gap increases with concentration of B and N. Higher field effect mobility. Higher current on/off ratio and resistive switching behavior.	Electrical properties depending upon thickness. Indirect band gap, semi‐conduction by double layered materials and direct band gap semi‐conduction by single layered materials with large gap. Low charge density, influenced by doping. Defects, electric field, mechanical strain, and surface adsorption influencing the electrical structures.	Direct, narrow, and sizeable band gap depending upon thickness. Moderate on/off ratio and higher carrier mobility High anisotropic band dispersion in the Brillouin zone (BZ) near the Fermi level. Functional and elemental functional group enabling to tune the electrical properties. Higher conductance and mobility along the arm‐chair direction.	Exhibiting excellent electrical properties with large band gap when functionalized. Band gap changing with the number of layers. Substitution, passivation, and functionalization enabling to tune band gaps. Superconductivity, insulative property via functionalization. Fluorination leading to Janus natured diamane, with both conduction and insulation properties.	Band gap and conductivity influenced by the film thickness and crystalline structure. Increased number of layers leading to decreased quantum confinement effect and direct band gap.
Magnetic properties	Not intrinsically magnetic. Both paramagnetism and ferromagnetism being reported, by introducing point defects, zig‐zag edges, and chemical doping. Vacancy, edge, and sp^3^ hybridization approach enabling localized magnetic moments.	Non‐magnetic. Functionalization, vacancy, or substitution of non‐magnetic impurities introducing magnetic properties and non‐zero magnetic moments. External strain stabilizing magnetic coupling, and hydrogen adsorption enhancing semi‐metallic and magnetic properties.	Unique electronic structure attractive for spintronics and valleytronics. Further experimental and theoretical studies needed.	Functional and elemental functional group (3D transition metal atoms) inducing the magnetism. Vacancies leading to magnetic properties.	Substitution of atoms opens the magnetic properties. Nitrogen (N) substitution resulting in ferromagnetic behavior; N vacancy leading to paramagnetic behavior. Electric field or desorption of functional groups leading to ferromagnetic behavior.	Too early to present, appearing closer to diamane, compared with graphene and its derivatives.
Optical Properties	Exceptional optical properties relating to fine structure constant, tunable infrared optical absorbance, and photo response in high frequency ranges. Affected by photon energy, doping agent, doping level, and absorption peak.	Minimum optical absorption, high transparency (99%), and 250–900 nm wavelength. Wide direct band gap and UV luminescence property providing sharp absorption at deep UV range. Strong photoluminescence and cathodoluminescence emissions in deep UV range, affected by planar faults. Hyperpolarizability.	Better photoresponsivity than other 2D materials. Switching behavior providing enhanced photo‐detection. Optical properties such as optical band gaps, mobility modifiable by the thickness of the sheets.	Thickness dependent band topology. Strong influence of linear dichronism, optical absorption, and relative orientation in photoresponse, Raman spectra, yield of photoluminescence emission, screening, and plasmonic effects. Arm‐chair direction exhibiting high degree polarization.	Wide band gap, optical peculiarities, resonance peak in valence band‐conductive band, and direct energy gap providing exceptional optical properties. Heterostructures of hydrogenated graphene and twisted bilayer graphene narrowing the mini bands inside the ultra‐wide 2D band gap to enhance optical properties.	Under investigation and concrete evidences under the way.
Thermal properties	Highest in‐plane thermal properties among all carbon.	Excellent thermal conductivity and stability. Favorable above 1600 K Superb high‐temperature resistance. Excellent dielectric characteristics.	Out‐of‐plane thermal conduction is low. Excellent thermoelectric characteristics. Grain sizes allowing for altering the thermal conductivity.	Poor thermal conductance but superconductive (3–16) K, originating from biaxial strain and doping.	Negligible thermal expansion at room temperature. AA stacked diamane exhibiting higher thermal conductivity than AB stacked diamane, due to horizontal stacked symmetry. Fluorinated and chlorinated diamanes being more thermally insulative than hydrogenated diamanes.	Good thermal conductivity, allowing for further improvement via dehydrogenation.
Mechanical properties	Excellent stiffness, outstanding mechanical strength, and high toughness. Strongest known material.	Mechanical robustness, dependent on the size and thickness. Low coefficient of friction. Exhibiting in‐plane piezoelectric behavior.	Good mechanical strength. Young modulus comparable to graphene oxide and steel. Semiconductor‐to‐metal transition under tensile and shear strain.	High tensile stress and strain, superb flexibility. Anisotropic mechanical response. Strong in‐plane anisotropy.	Hard material showing superior strength to graphene, decreasing with more layers. Good elasticity and tensile strength. High elastic modulus. Functionalization, and substitution further increasing the mechanical properties.	High strength, low coefficient of friction, and good bio‐compatibility. Further research is required.
Synthesis methods	Mechanical exfoliation of graphite by micromechanical cleavage (Scotch tape) method, mechanical milling, chemical reduction of graphene oxide, liquid phase exfoliation, electrochemical exfoliation, CVD, epitaxial growth, and arc evaporation of graphite under hydrogen.	Mechanical exfoliation, solvent‐assisted ultrasonication, acid exfoliation, chemical functionalization (noncovalent, ionic, covalent), unzipping of BNNT, and CVD,	Chemical exfoliation, PVD, ion exchange, thermolysis and powder vaporization, metal organic CVD, molecular beam epitaxy, and mechanical exfoliation.	Mechanical exfoliation, liquid phase exfoliation, lithiation, plasma‐assisted fabrication, pulsed laser deposition, high pressure method, CVD, and hydrothermal synthesis.	Computational method, CVD, application of pressure, substitution of various atoms as dopants, and Birch reduction.	Hot‐filament CVD.

## 6 Applications of Diamanes

### 6.1 Nanoelectronic, Optoelectronic, Thermo‐Electronic, and Quantum Devices

**Figure 12:**
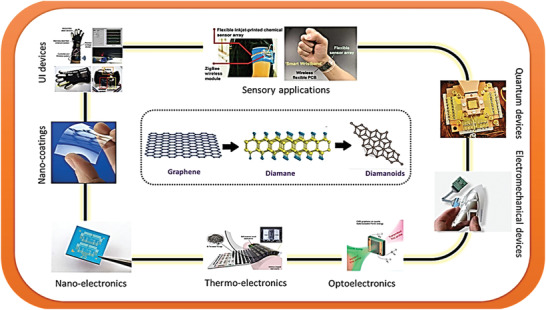
Probable applications (symbolic) of diamanes for new technologies of the near future.

The potential applications of diamanes in the nanotechnology and nanoengineering sector are vast and booming day by day.^[^ ^3^, ^4^, ^5^, ^6^, ^7^, ^8^, ^9^, ^10^ ^]^ Diamane has challenged the efficiency offered by Si to the semiconductor‐based industries, suggesting a worthy candidate to replace Si in the coming years.^[^ ^70^ ^]^ The extreme operating conditions of high temperature, high voltage, high‐frequency diamond thin films, or diamanes have been primarily evaluated by different groups for electronic applications. Excellent thermal conductivity and insulation both within a diamane in the presence of an external applied field could lead to self‐repair electronic devices (**Figure** **12**) particularly at extreme temperatures.^[^ ^85^ ^]^ The different application potentials of diamane in the field of nanoscience and nanotechnology are discussed in the following sections.

#### 6.1.1 Nano‐Electrical Devices

The most promising applications for diamane are identified in the electronic sectors. The bandgap and conductivity can be engineered by the deposition of functional groups and substitution of vacancies in graphene layers. The enhanced electrical characteristics from the nano level ranging from metallic to semiconducting behavior in the hydrogenated graphene remain at the frontline. Modifying the bandgap by changing the sp^2^ hybridization to sp^3^ hybridization could lead diamane towards real applications in nano‐electronic devices.^[^ ^83^ ^]^ Graphene functionalization opens the bandgap for electronic devices and circuit fabrication, due to the behavior of massless Dirac fermions and the exhibition of charge transport^.[^ ^101^ ^]^ In the case of transistor applications of semiconducting and nano‐electric devices, silicon dioxide (SiO_2_) is used as the back gate dielectrics and hexagonal BN flakes acts the top gate electrics which have been synthesized by using the AB stacked bilayered graphene.^[^ ^90^ ^]^

The electronic bandgap in diamanes enabled its applications in electronics such as field‐effect transistors, supercapacitors, lithium batteries, laser mediums, and other miniature devices.^[^ ^36^, ^37^, ^52^ ^]^ The functionalization by groups such as hydrogen, fluorine, and chlorine could equip diamane with semiconducting properties that are suitable for carbon‐based nanoelectronic devices (Figure 12). These functional groups are deposited in bilayered graphene via chemical vapor to increase the band gap.^[^ ^66^, ^67^, ^80^, ^86^ ^]^ Interestingly, simultaneous characteristics involving good electron mobility and hole mobility of fluorinated diamane, chlorinated diamane, and hydrogenated diamane have created a strong foundation for semiconducting and nano‐electrical applications.^[^ ^50^, ^70^, ^80^ ^]^ Moderate seed layer conditions under CVD to synthesize high‐quality, single‐crystal diamond films have been realized by fluorinating diamane.^[^ ^35^ ^]^ The carbon‐fluorine dipole formation in layers has paved the way to the fabrication of electronic and spintronic devices using these fluorinated diamanes.^[^ ^73^ ^]^ Insulating applications of nano‐electronics in tunnel contact could be fulfilled by functionalizing bilayered graphene with fluorine in the presence of hBN substrate. This created an insulating layer at the side where it was functionalized, whilst the other side remained conducting, due to the effective decoupling whereas double‐sided functionalization was fully insulating.^[^ ^73^ ^]^ The insulating characteristics of fluorinated diamane could be used in electron beam irradiation for nanopatterning technique, which selectively restores the AB‐stacked bilayer graphene. This technique may ultimately, become the building blocks for the fabrication of novel electronic devices, due to the wide bandgap depending on the surface termination species.^[^ ^35^ ^]^

Good hydrophobicity, low dielectric constant, low dissipation factor, and good optical properties ensure diamane a high place amongst many candidates for electronic packaging of fan‐out wafer‐level package.^[^ ^87^ ^]^ Small thickness and excellent electrical, optical, and mechanical properties spearhead their huge potential as the base of touch screen panels.^[^ ^31^ ^]^ Applications relating to field emission transistors and carbon‐based spintronics have also been proposed for diamanoid produced through pressure applications to bilayered graphene.^[^ ^46^ ^]^ TBG may also be used in nanoelectronics, due to the combined excellent electronic and mechanical properties, however detailed research on this topic is still lacking.^[^ ^68^ ^]^ Bilayered graphene with a 30˚ twist angle has led to the formation of fullerene‐like domains in the graphene mesh, which is suitable for applications in semiconducting hybrid films. High‐pressure applications in the bilayered and multilayered graphene without adsorbents could be utilized in nano‐heterostructures. For spintronics, semi‐hydrogenated diamanes with dangling bonds on the bottom layer arising with the periodic arrays have found use as a 2D ferromagnetic semiconductor with spin‐polarized bands.^[^ ^50^ ^]^ The ferromagnetic behavior of nitrogenated diamanes exhibiting a high concentration of holes, due to the large density in Fermi level, could be further explored in 2D magnetic semiconductors.^[^ ^39^ ^]^

#### 6.1.2 Thermo‐Electric Devices

Thermoelectric applications of diamane could take advantage of the property of both high thermal conductivity and low thermal conductivity in thermal management devices.^[^ ^36^, ^37^ ^]^ Wide band gap and ballistic thermal conductance have provided leverage for diamane to be used in thermal applications for electric devices. To obtain low thermal conductivity, heavy functional groups deposited materials can be selected, and vice versa.^[^ ^67^, ^86^ ^]^ The hydrodynamic characteristics due to the contribution of off‐plane acoustic modes and phonon transport enhanced the giant thermal conductivity, and eventually may find application in thermo‐electric systems such as tunable band gap semiconductors, field‐effect transistors, and so on.^[^ ^78^ ^]^ In a thermoelectric system, electricity and heat work side by side. Cooling and power generation systems from thermoelectric materials where the thermal difference is transferred to electrical energy or vice versa require good electrical conductivity and low thermal conductivity.^[^ ^63^ ^]^ The zero thermal expansion behavior on these carbon nanostructures is particularly attractive for applications where thermal strain is induced with a significant temperature fluctuation in the environment.^[^ ^70^ ^]^ The influence of stacking sequence and the functional groups is effective in the thermal conductivity, as the AA stacking exhibits better conductivity than in the AB stacking; and hydrogenated diamane has higher thermal conductivity than that of the fluorinated diamane, respectively. This can be due to the out‐of‐plane phonon mode in the hydrogenated diamane increasing the thermal conductivity, against the optical modes in fluorinated diamanes. The hydrogenated diamane is promising for applications where better heat conduction is required, whereas the fluorinated diamane is more suitable for those where better insulation is important.^[^ ^48^, ^84^ ^]^ The property of hydrophobicity of carbon‐fluorine bonds on the surface against non‐polar molecules enables the fluorinated graphene to a great heat transfer performance on Cu surface. Refrigeration property is seen which creates active sites and increases the bubble size once coated (Figure 12). Polymer coating to fluorinated graphene also enhances the chemical stability for harsh thermal cycling operations.^[^ ^87^ ^]^ Band engineering in 2D systems via doping and chemical modifications would further improve their performance,^[^ ^56^ ^]^ making them suitable for applications in thermo‐electrical systems and thermal management systems where both conductivity and insulation are required for smooth operations.

#### 6.1.3 Opto‐Electronic Applications

The development of flexible and transparent optoelectronic devices using 2D materials at a wafer‐scale is important, and functionalized diamanes offer such required properties.^[^ ^80^, ^81^ ^]^ Optic peculiarities and wide gap spectra with many resonance peaks in the valent and conductive bands in the density of states are required for nano‐optical and nanophotonic applications.^[^ ^50^ ^]^ The evidence of band gap, coinciding with the electronic and optical properties of diamane, is highly desirable for semiconductor devices and laser media in optoelectronic applications.^[^ ^1^, ^52^ ^]^ Phototonic devices (Figure 12) can be synthesized by using the AB stacked bilayered graphene with silicon dioxide (SiO_2_) and hexagonal BN acting as the back gate dielectrics and top gate electrics, respectively.^[^ ^90^ ^]^ Functional groups enhance the optical properties which are important in applications of optoelectronic devices. Enhancement of band gap by fluorine and chlorine offers the dynamic and thermal stabilities which are important for applications in photocatalysis.^[^ ^80^ ^]^ The narrow mini bands inside the ultra‐wide 2D band gaps of heterostructures of hydrogenated and Moire's diamane are promising for optoelectrical applications.^[^ ^47^ ^]^ Change in the sp^2^ to sp^3^ hybridization to open dielectric band gap regulates the applications of diamanes in nano‐optics.^[^ ^83^ ^]^ Irradiation caused by fluorinated diamane may be utilized in the nanopatterning technique, by restoring the AB stacked bilayered graphene in insulating films.^[^ ^35^ ^]^ Fluorinated diamanes have high mechanical stiffness, wide band gap for insulation, and direct energy gap for thin films, which are particularly attractive for nanophotonics.^[^ ^82^ ^]^ The characteristics of direct band gap, highly dispersed valence and conduction bands, and very small charge carrier effective masses enable the Janus C_4_HCl and C_4_FCl monolayers to be used in nanoelectronics and optoelectronics.^[^ ^59^ ^]^

Laser applications are important for various fields of optics, including medical uses, cutting and removal of stains, observation of light spectra, and other engineering applications. Active laser medium in diamanes can be obtained by controlling the number of layers, tuning the electrical properties, obtaining the thinnest dielectric properties, and other important characteristics.^[^ ^31^, ^36^, ^37^, ^51^, ^58^, ^72^ ^]^ Optical waveguides possess the properties of graphene dielectric nanostrips, superlattices, or integral waveguides.^[^ ^1^ ^]^ Optical planar waveguides, electron waveguides for electronic circuits, and resonance UV optical devices and elements can be formed by diamane and hydrogenation of twisted graphene.^[^ ^51^, ^58^, ^72^, ^78^ ^]^ Substitution of nitrogen and boron with carbon layer forms diamanes, which helps in the creation of active laser medium for ultimate application of optical waveguides.^[^ ^44^ ^]^ Creation of defects by adding dopants for tuning the electrical and optical properties in UTHD films can offer waveguide materials and photoelectronic materials, as it's low refractive index allows for fast speed of spread and low energy loss. Active layers in lasers can also be obtained by UTHD films.^[^ ^55^ ^]^ Tunnel devices, optical linear waveguides, and high‐frequency optoelectronic sensors are the future of the optoelectronic sectors, which can be fabricated by using heterostructures of graphene and diamanes.^[^ ^36^, ^37^ ^]^ Good electrochemical properties, light in weight, high in‐plane conductivity, and outstanding mechanical strength all favor the fluorinated graphene in energy applications.^[^ ^87^ ^]^ The graphite‐like diamond structures can be used in tunnel devices and optical waveguides due to the sandwich‐like structure. High‐efficient optoelectronic sensors, biosensors, lithium batteries, and supercapacitors could be constructed by taking advantage of gradient inter‐planar spacing of carbon materials.^[^ ^56^ ^]^ Thus, the enhanced optoelectrical properties offered by diamane could play an important part in the future nanotechnology.

#### 6.1.4 Quantum Devices

Quantum devices are very complex because technology has not reached far enough to thoroughly analyze the quantum behavior. Only a limited amount of work on diamane has been carried out in quantum environments. A recent theoretical study has revealed that diamanes and graphene can be used to study the quantum behavior. Moire's diamanes with diamond defects of nitrogen substituted vacancies and vacancy centers are important in developing quantum information processing.^[^ ^47^ ^]^ Single‐photon emission requires a host and needs precise prediction to optimal two‐level quantum systems (Figure 12). These predictions can be fulfilled by 2D materials using them as the sources for single photons. These sources could ultimately, find applications in quantum information processing.^[^ ^42^ ^]^ Configurations of qubits for the application in quantum computing is created by nitrogen‐vacancy (eV) in diamane, as a result, diamane could be further developed for such applications.^[^ ^37^ ^]^ Even though only a limited amount of research has been found in the quantum sector, scientists are trying hard to develop quantum‐related applications which is believed to be an interesting area of the future.

### 6.2 Electromechanical Applications

Mechanical tuning and thermal properties variation can be obtained by functionalization of bilayered graphene, to suit for electromechanical, nanoelectronics, and nanosensors applications.^[^ ^70^ ^]^ The number of layers in diamane does not have any influence on the mechanical strength, instead the zigzag directions create fractures independent of the tensile direction, and the strain/strength decreases with an increase in temperature.^[^ ^50^ ^]^ High hardness, excellent thermal conductivity, and wide band gaps make them promising for applications as electromechanical vibrators.^[^ ^50^ ^]^ Ultra‐thin dielectric layers of diamane with piezoelectricity, wide band gap are great candidate for constructing electromechanical system.^[^ ^1^ ^]^ For nanoelectronics, high mechanical stiffness is also a primary requirement.^[^ ^50^ ^]^ Functional groups influence the mechanical properties of diamanes. Large band gap opening and elasticity allows good electron mobility and hole mobility. These good electron mobility and hole mobility of both fluorinated diamane and hydrogenated diamane laid the foundation for electromechanical systems.^[^ ^50^, ^70^ ^]^ Active laser medium based on the enhanced properties of diamane is also useful in nanomechanical devices.^[^ ^58^ ^]^ The use of fluorinated graphene in composites could enhance the mechanical properties further, allowing to tailor the performance of the composites.^[^ ^87^ ^]^ Thinnest and hardest cutting tools, high‐performance electron emitter or circuit board could be another application of the 2D diamond.^[^ ^56^ ^]^ Overall, mechanical properties are important in virtually all nanotechnological devices, as strength and ductility are always crucial to affect the reliability and performance in applications.

### 6.3 Sensor

Sensor applications of diamane and its family are increasing at a very fast pace. The excellent vibrational and resonance properties enable their sensory applications (Figure 12). Ultra‐sensitive resonator‐based nanosensors using diamane resonators have been demonstrated, by exploiting it's high natural frequency and quality factors.^[^ ^87^ ^]^ Excellent resonating and vibrational characteristics of diamane nanoribbon resulted in less intrinsic energy dissipation during oscillation. The natural frequency, quality factor, figure of merit, and in‐plane stiffness are influenced by the pre‐tensile strain. These characteristics can in turn be explored as a mechanical resonator in various sensing applications with precise sensitivity and high efficiency.^[^ ^36^, ^37^, ^48^, ^88^ ^]^ Hydrogen functionalized diamane has already been reported as a mechanical resonator.^[^ ^59^ ^]^ Thermal conductivity due to the consequence of hydrodynamic character could be exploited in nano‐magnetic resonance imaging and sensing resonators.^[^ ^78^ ^]^ When silicon vacancy is created to diamanes, it introduces impurities to the wide band gap, which enables applications in photodetection and photocatalysis in the IR/visible region.^[^ ^77^ ^]^ Dopant substituted diamane will also bring in new properties attractive for resonating devices. Nitrogen substitution during the fabrication enhanced diamane by creating a model framework for band gap reduction. This reduction decreases the electrical property, however it simultaneously increases the magnetic and vibrational properties, which are attractive for nano‐magnetic resonance imaging. Their spin nitrogen‐vacancy centers in nano‐electronics and sensory applications could offer enhanced efficiency than the vacancy centers of bulk diamond.^[^ ^41^, ^44^ ^]^ Diamane formed by TBG has been used in magnetic resonance imaging, based on its vibrational and resonance properties.^[^ ^78^ ^]^ Moire's diamanes with diamond defects of nitrogen substituted vacancies and vacancy centers are also great candidate for applications in magnetic, electrical, and temperature sensor engineering.^[^ ^47^ ^]^ Diamane also demonstrated great electrochemical sensing properties. Hydrogenation, fluorination, and oxygenation of diamane could be developed for chemical sensors and biosensors,^[^ ^99^ ^]^ to detect uric acid, explosives, ascorbic acid, and dopamine by using partially hydrogenated graphene and graphene alone in biomarkers. The sensing of hydrogen evolution and oxygen reduction over bare glassy carbon electrode surfaces has been documented by using diamanes. The sensitivity for detecting ethanol, methane, and formaldehyde are reportedly enhanced, with excellent selectivity for the separation of carbon dioxide (CO_2_) from nitrogen (N_2_).^[^ ^63^, ^87^ ^]^ Nitrogen and boron doping formed diamane has nanometer dielectrics that can be tuned through doping, which is an appealing character for chemical sensors. Thus, the field of sensory development based on diamanes is equally interesting.

### 6.4 Coatings and Lubrications

**Figure 13:**
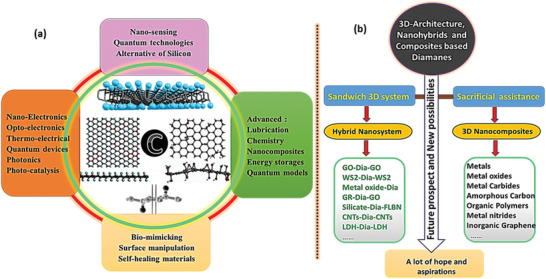
a) Outline about the cutting‐edge research and innovative technological applications of various diamanes and their derivatives for the next generation of multifunctional devices. b) Diagrammatic representation of a roadmap for the development of diamanes and diamanoids as a major constituent in new 3D nanomaterials and nanocomposites.

Diamanes can provide various coating materials with superb strength for applications where wear and tear occurs. Diamane formed after shear‐driven phase transformation of graphene could be utilized in the defense and coating industries.^[^ ^93^ ^]^ Hydrogenated diamanes have a low coefficient of friction, which is particularly attractive as ultra‐thin coatings on mechanical parts to improve tribological performance. Composite and aerospace applications requiring ultra‐high strength and improved stiffness, could be offered by these hydrogenated diamanes.^[^ ^36^, ^37^, ^48^, ^105^ ^]^ Super hard coating materials derived from the nitrogen substituted diamanes are specifically suitable to wear, tear, and waterproofing.^[^ ^39^ ^]^ Lubrication has long been an granted area of the graphitic materials and carbon structures. New additives such as hBN and other graphitic materials would bring in new life for the further development of advanced tribological coatings. Graphene films have been reported to decrease the friction coefficient between friction pairs.^[^ ^85^, ^87^ ^]^ The application of diamane in the tribology sectors needs intensive research. The current cutting‐edge research status of diamane, diamanoids, and similar carbon nanosystems is presented in **Figure** **13**a. In addition, for better understating, a roadmap for the future prospect of diamane and diamanoids is also schematically presented as Figure 13b. We believe that within 10 years, some of these research work predicted in Figure 13b will be materialized.

## 7 Applications of Diamanoids

In the family of nano carbons, diamanoids are the youngest member, and only 20–25 research papers associated with them (theoretical and experimental investigation) have been published. Therefore, it is too early to describe their applications. To date, 2D carbon nanomaterials have yet to convincingly demonstrate their real technological applications, and researchers around the globe are trying hard to convert hopes into reality. The outstanding properties of diamanoids, such as the wide band gaps, high thermal conductivity, high radiation, and great chemical inertness, high C—C bond energies, and negative electron affinity, are surely an appealing asset for the development of new electronic devices, dry batteries, asymmetric supercapacitors, and advance composites, especially for applications under harsh conditions.^[^ ^48^, ^50^, ^52^, ^53^, ^61^ ^]^ In this context, a robust cathode system could be an example area for using cold diamanoids in the satellites (space applications), where very high resistive and high‐power travel waves are needed, on top of tribological properties.^[^ ^69^, ^70^, ^72^, ^73^, ^74^, ^75^, ^76^, ^77^ ^]^ It has also been suggested that diamanoids could be a better alternative to graphene and graphane for data storage (in nano‐device), by using nitrogen dioxide, nitric oxide, and silicon radicals as the additional component.^[^ ^89^ ^]^ Solid lubrication for high‐temperature engineering, ultra‐thin protective coatings, active media for lasers in nano‐optics, and thermal management devices are all possible field of research, based on their high thermal stability and low coefficient of friction.^[^ ^80^, ^81^, ^82^, ^83^, ^84^, ^85^ ^]^ Boldly speaking, based on the fundamental features of diamanoids, diamanoids would find applications as excellent wear‐resistant coating and anti‐adhesion environments.^[^ ^53^ ^]^

Recently, dopant substituted diamanoids for quantum computing applications have been investigated. The 2D layered morphology, ultra‐thinness, high chemical stability, and biocompatibility with low friction coefficient make the diamanoids an ideal candidate for biomedical engineering.^[^ ^38^ ^]^

## 8 Future Prospects

Whenever a new material is discovered, enough fundamental and engineering research is needed prior to identifying meaningful applications. Diamane and diamanoids are in their infancy, being discovered only recently, therefore intensive studies to fully understand them are urgently required. Early preliminary research on diamane and diamanoids has created a high hope about their appropriateness for quantum‐electronic, quantum‐computing, nano‐sensors, next generation energy storage devices, etc. as discussed in the above sections. However, various challenges are prominent too, in exploring the advanced chemistry, physics, and quantum characteristics, especially for the controlled and effective tuning of the stability, electronic properties, and scaling up process, for practical use. In this context, we highlight a few crucial challenges that we consider them as the main bottleneck for the development of diamane, diamanoids, and similar ultra‐thin carbon films.

1. Diamane and its derivatives have been tested in wearable devices, conducting textiles, electronics, photonics, sensors, energy storage, harvesting, environmental barriers, and soft actuators. These applications need to be improved through cost‐effective synthesis and efficient surface manipulation. Earlier, CNTs, graphene, etc. have been thoroughly investigated for similar purpose, the research inputs and outputs from diamanes are very demanding.^[^ ^74^ ^]^
2. Graphene and graphene oxide‐based light‐emitting diodes, plasmon resonance for photocurrent in applications of high‐speed optical devices, foldable touch screens, supercapacitors, and other fascinating devices have been advanced. These devices need further engineering and tuning to get robust performance by using diamane and diamanoids.^[^ ^72^ ^]^
3. Synthesis of diamane and diamanoids from graphene is a tedious, expensive, and time taking method. Nowadays, the production of graphene and similar nanocarbons from biomass precursor is an emerging field. A green production technique for diamane and diamanoids from biomass could be an exciting area of research, contributing to the development of a sustainable society.
4. An alternative route for the formation of diamane/diamanoids using hydrogen flux without the requirement of high pressure is possible. These conditions exist in space and can be seen through the Spitzer space telescope, which could be considered for further investigation on the galactic formation.^[^ ^36^ ^]^ In‐depth investigation could lead to the bulk production of diamane/diamanoids.
5. Diamane and diamanoids can be used as a heat‐resistant coating on the space shuttle and other aerospace equipment, to withstand a tremendous amount of heat and significant temperature fluctuations.
6. The configuration of diamond‐like ultrathin films can be tuned to enhance different properties, but the precise control over the extent of structural modifications poses a gigantic challenge for achieving excellent thermo‐mechanical properties.^[^ ^65^ ^]^ Tuning these properties at the atomic level to manipulate the band gap has opened a new door for hydrogen storage in 2D nanomaterials, color‐changing lasers, and self‐arranging electronic circuits. Surface engineering of diamane is therefore, another very promising area, to achieve better efficiencies for those applications.
7. The role of functional groups in tuning electronic and thermal conduction of diamanes must be fully investigated to completely appreciate the potentials they have offered, such as for the fabrication of new lightweight zero thermal expansive materials and related devices.^[^ ^59^, ^70^ ^]^ In particular, superconductivity investigation of the metal‐diamond structure is another area of interest, as the diamagnetic electronic structure of diamane appears to be very intriguing.^[^ ^60^ ^]^
8. The research on quantum behavior, electron tunneling, and electronic bandgap calculations of diamane and diamanoids is very limited. This field of research is intriguing and mesmerizing but due to the lack of pace in research, newer innovation using quantum parameters has yet to be produced.^[^ ^52^ ^]^
9. Mutilated layered diamane with sandwiched metal nanoparticles on a suitable substrate is another direction related to diamane and diamanoids research. Novel ultra‐thin carbon materials with sp^3^–sp^2^ carbon hybridization could bring in new properties.^[^ ^43^, ^60^ ^]^
10. Cold diamanoid cathodes have been used to power satellites because these electrodes can sustain harsh environmental conditions without affecting applicability and portability. Therefore, more research in this area is very important for the use of diamane and diamanoids in space exploration.^[^ ^89^ ^]^

Based on these issues, we believe that diamanes and diamanoids have great potentials in the future for a variety of engineering applications. Using advanced tools of nanotechnology with the assistance of DFT simulations, we could turn diamanes and diamanoids to be the ideal nanomaterials for quantum computing and quantum electronics. If most of these challenges were tackled within the next 10 years, the scientific community would be able to lay a solid new foundation for the exploitation of diamanes and diamanoids toward more practical applications.

## 9 Conclusions and Outlook

This review paper presents a detailed overview of the recent progress in the fabrication, atomic structure, physical properties (such as electronic, optical, thermal, mechanical, and vibrational peculiarities), and cutting‐edge technological applications of diamanes and diamanoids. The first section of this review describes the historical prospects, structural characteristics, and physical properties. In the second section, we discussed the types of diamane, methods of synthesis, doping, and functionalization to manipulate its surface properties. Special attention was focused on the band gap manipulation, surface modification, hetero atom/metal doping, and quantum mechanical properties of diamanes. Moreover, potential applications of diamanes and diamanoids for nanoelectronic, semiconducting, optoelectronic, quantum electronic, etc. have been highlighted. In the third section, a brief overview of the synthesis of diamanoids and point wise plausible use of diamane in different nanodevices has been described. Current research updates, plain explanations, and appropriate bibliography for further reading provided in this article could open a door for readers to convert a predesigned Van der Waals layered precursor into a new covalently bonded layered nanosystem with desired properties, for certain applications. In the final section, future prospects for diamane‐ and diamanoid‐based technology have been discussed. We hope that this review could promote further research and innovation on diamanes, diamanoids, and other ultrathin films.

## Conflict of Interest

The authors declare no conflict of interest.
